# Potentially inappropriate prescribing in polymedicated older adults with atrial fibrillation and multimorbidity: a Swedish national register-based cohort study

**DOI:** 10.3389/fphar.2024.1476464

**Published:** 2024-09-10

**Authors:** Cheima Amrouch, Davide Liborio Vetrano, Cecilia Damiano, Lu Dai, Amaia Calderón-Larrañaga, Maxim Grymonprez, Marco Proietti, Gregory Y. H. Lip, Søren P. Johnsen, Jonas W. Wastesson, Kristina Johnell, Delphine De Smedt, Mirko Petrovic

**Affiliations:** ^1^ Department of Public Health and Primary Care, Ghent University, Ghent, Belgium; ^2^ Department of Internal Medicine and Paediatrics, Ghent University, Ghent, Belgium; ^3^ Department of Neurobiology, Aging Research Center, Care Sciences and Society, Karolinska Institutet and Stockholm University, Stockholm, Sweden; ^4^ Stockholm Gerontology Research Center, Stockholm, Sweden; ^5^ Department of Cardiovascular, Endocrine-Metabolic Diseases and Aging, Istituto Superiore di Sanità, Rome, Italy; ^6^ Department of Bioanalysis, Pharmaceutical Care Unit, Ghent University, Ghent, Belgium; ^7^ Department of Clinical Sciences and Community Health, University of Milan, Milan, Italy; ^8^ Division of Subacute Care, IRCCS Istituti Clinici Scientifici Maugeri, Milan, Italy; ^9^ Liverpool Centre for Cardiovascular Science at University of Liverpool, Liverpool John Moores University and Liverpool Heart and Chest Hospital, Liverpool, United Kingdom; ^10^ Danish Center for Health Services Research, Department of Clinical Medicine, Aalborg University, Aalborg, Denmark; ^11^ Department of Medical Epidemiology and Biostatistics, Karolinska Institutet, Stockholm, Sweden

**Keywords:** polypharmacy, atrial fibrillation, inappropriate prescribing, STOPP/START, adverse health outcomes

## Abstract

**Introduction:**

Current research on potentially inappropriate prescribing (PIP) in polymedicated older adults with atrial fibrillation (AF) and multimorbidity is predominantly focused on PIP of oral anticoagulants (OAC). Our study aimed to assess (i) the overall prevalence of PIP in older multimorbid adults with AF, (ii) potential associated factors of PIP, and (iii) the association of PIP with adverse health outcomes in a nationwide sample of Swedish older adults.

**Methods:**

Swedish national registries were linked to establish a cohort with a 2-year follow-up of older adults (≥65y) who, on 1 January 2017, had a diagnosis of AF and had at least one comorbidity (n = 203,042). PIP was assessed using the reduced STOPP/START version 2 screening tool. The STOPP criteria identify potentially inappropriate prescribed medications (PIM), while the START criteria identify potential prescribing omissions (PPO). PIP is identified as having at least one PIM and/or PPO. Cox regression analyses were conducted to examine the association between PIP and adverse health outcomes: mortality, hospitalisation, stroke, bleeding, and falls.

**Results:**

PIP was highly prevalent in older adults with AF, with both polypharmacy (69.6%) and excessive polypharmacy (85.9%). In the study population, benzodiazepines (22.9%), hypnotic Z-medications (17.8%) and analgesics (8.7%) were the most frequent PIM. Anticoagulants (34.3%), statins (11.1%), vitamin D and calcium (13.4%) were the most frequent PPO. Demographic factors and polypharmacy were associated with different PIM and PPO categories, with the nature of these associations differing based on the specific type of PIM and PPO. The co-occurrence of PIM and PPO, compared to appropriate prescribing, was associated with an increased risk of adverse health outcomes compared to all appropriately prescribed medications: cardiovascular (CV) (Hazard ratio (HR) [95% confidence interval] = 1.97 [1.88–2.07]) and overall mortality (HR = 2.09 [2.03–2.16]), CV (HR = 1.34 [1.30–1.37]) and overall hospitalisation (HR = 1.48 [1.46–1.51]), stroke (HR = 1.93 [1.78–2.10]), bleeding (HR = 1.10 [1.01–1.21]), and falls (HR = 1.63 [1.56–1.71]).

**Conclusion:**

The present study reports a high prevalence of PIP in multimorbid polymedicated older adults with AF. Additionally, a nuanced relationship between prescribing patterns, patient characteristics, and adverse health outcomes was observed. These findings emphasise the importance of implementing tailored interventions to optimise medication management in this patient population.

## Introduction

Worldwide, atrial fibrillation (AF), the most prevalent arrhythmia, affected 8.8% of the population aged 75 years or older, in 2019 (Global Burden of Disease Collaborative Network, 2020). The incidence and prevalence of AF increases with age (Lippi et al., 2021; Chen, 2016; Zhang et al., 2021; Kornej et al., 2020). Moreover, AF is associated with an increased risk of heart failure, stroke, bleeding, mortality, and substantial healthcare costs (Kornej et al., 2020; Buja et al., 2024; Burdett and Lip, 2022).

Older adults with AF often have a clinically complex profile (Romiti et al., 2022), presenting with multiple health conditions (multimorbidity) and, consequently, polypharmacy (concomitant use of ≥5 medications) (Kozieł et al., 2021; LaMori et al., 2011; Dalgaard et al., 2020; Proietti et al., 2019). Multimorbidity and polypharmacy contribute to the clinical complexity of AF patients, with implications for the treatment and prognosis (Proietti et al., 2020; Kannel et al., 1982; Grymonprez et al., 2024; Zheng et al., 2023), as well as increasing susceptibility to potentially inappropriate prescribing (PIP) (Payne et al., 2014). PIP involves prescribing medications that (i) may pose more risk than benefit, (ii) lack an evidence-based indication, or (iii) are potentially inappropriately omitted (O’Connor et al., 2012). Moreover, PIP is associated with adverse drug events, hospitalisation, increased healthcare costs, morbidity, and higher mortality, highlighting the need for vigilant monitoring of prescribing practices and interventions (Gallagher et al., 2020; Guaraldo et al., 2011; Lozano-Montoya et al., 2015; Lucchetti and Lucchetti, 2017; Wallace et al., 2017; Tommelein et al., 2015; Moriarty et al., 2019; Robinson et al., 2022). Current research on PIP in multimorbid and polymedicated older adults with AF is predominantly focused on PIP of (direct) oral anticoagulants ((D)OAC), and little evidence exists regarding the PIP of other medications (Amrouch et al., 2024).

Our study aimed to: (i) assess the overall prevalence of PIP in older adults with AF and multimorbidity, stratified by polypharmacy levels; (ii) investigate demographic characteristics and polypharmacy as potential associated factors of PIP; and (iii) quantify the association of PIP with adverse health outcomes using the Swedish administrative health registries.

### Methodology

The present study was performed as part of the Atrial fibrillation integrated approach in frail, multimorbid and polymedicated older people Horizon 2020 project (AFFIRMO, grant agreement n. 899871). AFFIRMO aims to develop, implement, and assess the effectiveness of a patient-centred, holistic, and integrated care strategy based on the ‘Atrial Fibrillation Better Care’ ABC model (Johnsen et al., 2022).

### Study design and participants

This study consists of a national register-based cohort comprising Swedish adults. Adults with a diagnosis of AF and at least one comorbidity between 1 January 2012, and 31 December 2016, and aged 65 years or older at baseline (1 January 2017) were included. The cohort was followed up for a period of 2 years (1 January 2017 - 1 January 2019). Drug dispensing data for the 90 days preceding baseline were collected. Ethical approval was granted by the Regional Ethical Review Board of Stockholm (dnr: 016/1001–31/4, 2020–03525; 2021–02004).

To establish the cohort, individual-level data from various Swedish national health registries were linked through the unique personal identification number and pseudonymised to the researchers, including the Swedish Total Population Register, National Education Register, National Patient Register, National Prescribed Drug Register, and the National Cause of Death Register^1^.

### Data sources and variables

The Total Population Register, managed by Statistics Sweden, has been gathering life events data since 1968, from which information on age, sex, individual-based income, and civil status was extracted (Ludvigsson et al., 2016). The Swedish National Education Register documents the highest level of formal education attained by individuals, ranging from elementary to post-graduate level^2^.

The National Patient Register is a health register, recording in-patient admissions since 1987 and out-patient specialist visits since 2001^3^. Primary care data are not included in this register. Extracted variables for this study included the date of hospital admission and discharge (for hospital admissions) and the diagnoses made during the hospitalisation or specialist visit. Diagnoses are coded using the International Classification of Diseases version 10 (ICD10). The chronic disease categories were derived from the recorded diagnoses (Calderon-Larranaga et al., 2017).

The National Prescribed Drug Register, established in July 2005, records pharmacy-dispensed medication prescriptions nationwide^4^. Medications are categorised using the Anatomical Therapeutic Chemical (ATC) classification. This register does not encompass medications administered in hospital settings, vaccines, and over-the-counter medications.

The National Cause of Death Register, documents all deaths electronically since 1952, including the date and causes of death using ICD10 codes (Socialstyrelsen, 2024).

This study focused on various adverse health outcomes, including overall causes of hospitalisation and death, cardiovascular (CV) causes of hospitalisation and death, bleeding, stroke, and injurious falls. Additional file A0 provide the ICD10 codes defining these outcomes.

### Potentially inappropriate prescribing

The Screening Tool of Older Persons Prescriptions/Screening Tool to Alert doctors to Right Treatment (STOPP/START) version 2 (v2) medication screening tool was used to evaluate PIP (Mahony et al., 2018). STOPP consists of 80 criteria aimed at identifying potentially inappropriate prescribed medications (PIM), such as medications that should be avoided in older adults because the risk outweighs the benefit, doses that should not be exceeded and medications contraindicated for specific conditions. START consists of 34 criteria and identifies potential prescribing omissions (PPO). These are medications that are omitted from the treatment scheme but that should be prescribed (i.e., medications which are clinically indicated for a patient but are not prescribed). The STOPP/START criteria are organised in categories according to physiological systems.

The operationalisation of the reduced STOPP/START v2 criteria was based on the work of Huibers et al., 2019 (Additional file A1) (Huibers et al., 2019). To enable the automated application of the reduced STOPP/START v2 criteria, several assumptions and adjustments were necessary (Additional file A2). In the present data source, 65% (52/80) of the STOPP criteria and 59% (20/34) of the START criteria were applicable. None of the START categories related to *Indication of medications*, *Urogenital system* and *Vaccines* were applicable. A comprehensive overview can be found in additional file A2.

### Statistical analysis

Categorical variables were presented as frequencies and percentages, while continuous variables were expressed as medians with interquartile ranges [IQR]. Statistical comparisons between proportions and non-parametric continuous variables were conducted using Chi-square tests and the Kruskal–Wallis Rank Sum test, respectively.

Multinomial logistic regression was used to identify demographic and clinical factors associated with PIP, presented as adjusted odds ratio (ORs) with 95% confidence interval (95%CI). PIP was categorised into four levels: *appropriate prescribing (reference group),* presence of only STOPP criteria (PIM), presence of only START criteria (PPO), and presence of both STOPP and START criteria (PIM&PPO). Unadjusted and adjusted multivariable binary logistic regression models were used to assess factors associated with the most prevalent reduced STOPP/START v2 categories (>5%), presented as ORs with 95%CI.

Cox proportional hazard regression models, unadjusted and multivariable adjusted, were fitted to examine the association between PIP, prevalent PIM/PPO categories and adverse health outcomes observed over a 2-year follow-up period. The results were presented as hazard ratio (HR) with 95%CI. These outcomes were examined independently and included overall and CV mortality, overall and CV hospitalisation, stroke, bleeding, and injurious falls. The reference group for the examination of PIP, PIM and PPO categories consisted of individuals with all medications appropriately prescribed.

Models were adjusted for demographic variables (sex, age, civil status, education, and income) and polypharmacy. Age was categorised into three groups: *young old (65 - < 75 years) (reference group),* old (75 - < 85 years) and oldest old (≥85 years). Civil status was categorised into two levels: *married/partnered (reference group)* and unpartnered (unmarried/divorced/widow). Education was differentiated into three levels: *elementary (reference group),* high school, and university or higher. Income data from 2016 was categorised into quintiles (Q) *(reference group: income Q1).* Polypharmacy was categorised into three levels: *no polypharmacy (<5 medications) (reference group),* polypharmacy (five to nine medications) and excessive polypharmacy (≥10 medications).

Data were assessed for multicollinearity (cut-off generalised variance-inflation factor >5), resulting in the exclusion of the number of diseases as a covariate (Fox and Monette, 1992; Vatcheva et al., 2016). Statistical significance was defined as p-value <0.05. Data analyses were performed using R version 4.3.2 and RStudio version 2024.04.0 + 735.

## Results

### Demographic characteristics

This Swedish national register-based cohort study consisted of 203,042 persons aged 65 years or older with AF and at least one comorbidity. The median age of the study population was 79.4 years [IQR 73.2–85.6] with 55.2% of the cohort being male (Table 1). The median number of drugs taken was 7 [4–10] and the median number of diseases diagnosed was 6 [4–8]. Most (72.9%) were prescribed five or more medications. OAC were prescribed for 64.0% of the population, with 31.9% of the total population receiving specifically a direct OAC (DOAC).

**TABLE 1:** Descriptive characteristics of the population at baseline.

Variables	Total n = 203,042	No polypharmacy (<5 )n = 54,943	Polypharmacy (5–9) n = 95,212	Excessive polypharmacy (≥10) n = 52,887
Age, median [IQR]	79.4 [73.2–85.6]	76.7 [71.5–83.2]	79.8 [73.6–85.9]	81.3 [74.7–87.0]
Female, n (%)	91027 (44.8%)	20324 (37.0%)	42889 (45.0%)	27814 (52.6%)
Age groups (years), n (%)				
65–74 years	66100 (32.6%)	23633 (43.0%)	29104 (30.6%)	13363 (25.3%)
75–84 years	81231 (40.0%)	20641 (37.6%)	38764 (40.7%)	21826 (41.3%)
+85 years	55711 (27.4%)	10669 (19.4%)	27344 (28.7%)	17698 (33.5%)
Education, n (%)				
Elementary	82743 (40.8%)	18993 (34.6%)	39266 (41.2%)	24484 (46.3%)
High school	76122 (37.5%)	21155 (38.5%)	35651 (37.4%)	19316 (36.5%)
University or more	44177 (21.8%)	14795 (26.9%)	20295 (21.3%)	9,087 (17.2%)
Civil status, n (%)				
Married/partnered	97352 (47.9%)	29865 (54.4%)	45979 (48.3%)	21508 (40.7%)
Unpartnered	105690 (52.1%)	25078 (45.6%)	49233 (51.7%)	31379 (59.3%)
Income, n (%)				
Q1	39726 (19.6%)	9,583 (17.4%)	18998 (20.0%)	11145 (21.1%)
Q2	40622 (20.0%)	9,026 (16.4%)	18978 (19.9%)	12618 (23.9%)
Q3	40880 (20.1%)	9,829 (17.9%)	19081 (20.0%)	11970 (22.6%)
Q4	40888 (20.1%)	11861 (21.6%)	19406 (20.4%)	9,621 (18.2%)
Q5	40926 (20.1%)	14644 (26.7%)	18749 (19.7%)	7,533 (14.2%)
Number of medications, median [IQR]	7 [4–10]	3 [2–4]	7 [6–8]	12 [11–14]
Number of diseases, median [IQR]	6 [4–8]	4 [3–6]	6 [4–8]	9 [6–11]
OAC (VKA^1^ or DOAC^2^) use, n (%)	130040 (64.0%)	28569 (52.0%)	65654 (69.0%)	35817 (67.7%)
DOAC use, n (%)	64782 (31.9%)	14829 (27.0%)	31876 (33.5%)	18077 (34.2%)
Antiplatelet^3^ use, n (%)	24690 (12.2%)	3,227 (5.9%)	11582 (12.2%)	9,881 (18.7%)

DOAC, direct oral anticoagulants; IQR , interquartile range [IQR]; OAC, oral anticoagulants; VKA, vitamin K antagonists; Q = quintile, ^1^ATC code for VKA, B01AA; ^2^ATC codes for DOAC, B01AE07; B01AF01; B01AF02; and B01AF03; ^3^ATC code for antiplatelets B01AC.

### Prevalence of PIP

Most of the study population (73.2%) had at least one PIM or PPO. In 14.5% of the population, only PIM were present without PPO, while in 33.9% of the population only PPO were identified without PIM. A total of 50,195 individuals (24.7%) had at least one PIM and at least one PPO (PIM&PPO) (Additional file A3: Supplementary Table S1).

**FIGURE 1:**
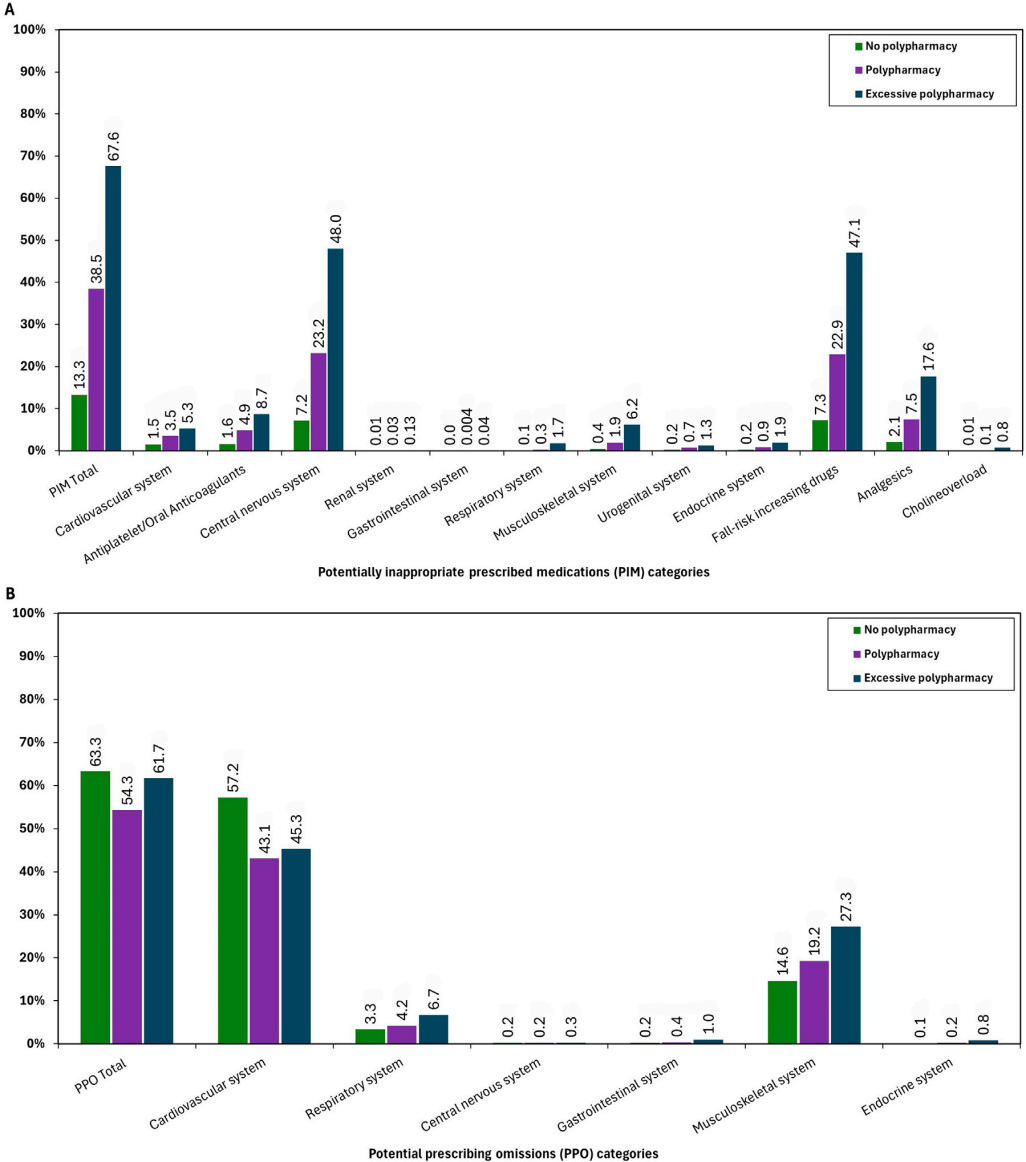
Prevalence of the reduced STOPP (PIM)/START(PPO) criteria version 2 categories stratified by polypharmacy levels. *PIM= potentially inappropriate prescribed medications, PPO = potential prescribing omissions*.

Among the STOPP (PIM) categories, Central nervous system (CNS) drugs *(benzodiazepines)* (25.3%) and Fall Risk Increasing Drugs (FRIDs) (*benzodiazepines, hypnotic Z-drugs)* were highly prevalent (25.0%) (Additional file A3: Supplementary Table S1; Figure 1A). Analgesics were potentially inappropriately prescribed in 8.7% of the population. CV medications *(β-blocker)* and antiplatelet/anticoagulants (AP/OAC) were potentially inappropriately prescribed in respectively 3.4% and 5.0% of the study population. The other PIM categories occurred in less than 3.0% of the overall study population. Older adults with excessive polypharmacy consistently exhibited a higher prevalence of PIM compared to those with no polypharmacy and polypharmacy (Figure 1A).

Among the START (PPO) categories, CV drugs *(OAC, statins, β-blocker and Angiotensin Converting Enzyme (ACE) inhibitors)* (47.5%) and musculoskeletal (MSK) system drugs *(vitamin D, calcium and bisphosphonates)* (20.1%) were highly prevalent (Supplementary Table S1; Figure 1B). Specifically, in 34.3% of the population, no OAC were prescribed *(START criterion A1),* while in 25.9% of the population neither AP nor OAC were prescribed *(STARTA2 criterion).* The prevalence of other PPO categories was less than 5.0% in the overall population.

### Factors associated with PIM, PPO and prevalent categories

Polypharmacy and excessive polypharmacy were significantly associated with higher odds of experiencing PIM, PIM&PPO, and prevalent PIM categories compared to individuals without polypharmacy, after multivariable adjustment (Table 2, 3). In contrast, patients with (excessive) polypharmacy showed lower odds of PPO and CV PPO, while exhibiting higher odds of MSK PPO.

**TABLE 2:** Association (OR and 95%CI) between demographic factors and potentially inappropriate prescribing. Estimates are derived from multinomial regressions.

	PIM OR [95%CI]	PPO OR [95%CI]	PIM&PPO OR [95%CI]
**Age**			
75–84 years	1.21 [1.17–1.26]	1.20 [1.17–1.23]	1.42 [1.37–1.46]
≥85 years	1.60 [1.53–1.67]	1.62 [1.57–1.68]	2.29 [2.21–2.37]
**Sex**			
Male sex	0.77 [0.75–0.80]	1.14 [1.11–1.17]	0.88 [0.86–0.91]
**Education**			
High school	1.06 [1.02–1.09]	*1.02 [0.99–1.04]*	1.06 [1.02–1.09]
University or more	1.14 [1.09–1.19]	0.96 [0.93–0.99]	*1.03 [0.99–1.07]*
**Civil status**			
Unpartnered	1.14 [1.10–1.17]	1.2 [1.17–1.23]	1.37 [1.33–1.41]
**Income**			
Q2	*1.02 [0.97–1.07]*	*0.98 [0.94–1.02]*	*1.04 [0.999–1.09]*
Q3	*1.03 [0.98–1.07]*	0.96 [0.92–0.99]	1.05 [1.01–1.10]
Q4	*1.00 [0.95–1.05]*	0.89 [0.85–0.92]	0.91 [0.88–0.86]
Q5	*1.02 [0.97–1.08]*	0.81 [0.78–0.84]	0.82 [0.79–0.86]
**Polypharmacy status**			
Polypharmacy	4.04 [3.84–4.24]	0.59 [0.57–0.60]	2.37 [2.29–2.46]
Excessive polypharmacy	13.34 [12.64–14.08]	*0.72 [0.70–0.75]*	9.06 [8.69–7.44]

*CI, confidence interval; OR, odds ratio; PIM, potentially inappropriate prescribed medications; PPO, potential prescribing omissions; PIM&PPO*, *having at least one PIM, and one PPO., The reference groups are: All appropriately prescribed medications, age range [65–74] years, female sex, elementary education, having a partner, lowest income quintile (Q1), and no polypharmacy*. The numbers in Italic suggest that the values were statistically nonsignificant *p* > 0.05.

**TABLE 3:** Association (OR and 95%CI) between demographic factors and each reduced STOPP (PIM)/START (PPO) category with prevalence >5% and cardiovascular PIM. Estimates are derived from adjusted binary logistic regression models.

	CV PIM OR [95%CI]	AP/OAC PIM OR [95%CI]	CNS PIM OR [95%CI]	FRIDs PIM OR [95%CI]	Analgesics PIM OR [95%CI]	CV PPO OR [95%CI]	MSK PPO OR [95%CI]
**Age**							
75–84 years	1.22 [1.15–1.30]	0.90 [0.86–0.95]	1.33 [1.30–1.37]	1.36 [1.33–1.40]	0.84 [0.81–0.88]	1.20 [1.17–1.22]	1.17 [1.13–1.20]
≥85 years	1.26 [1.18–1.35]	0.67 [0.64–0.71]	1.86 [1.81–1.92]	1.99 [1.93–2.05]	0.83 [0.80–0.87]	1.49 [1.45–1.52]	1.67 [1.62–1.72]
**Sex**							
Male sex	1.18 [1.11–1.24]	1.42 [1.35–1.49]	0.64 [0.63–0.66]	0.66 [0.64–0.67]	0.81 [0.78–0.83]	1.25 [1.23–1.28]	0.76 [0.74–0.78]
**Education**							
High school	1.07 [1.01–1.13]	*1.04 [0.99–1.09]*	1.06 [1.04–1.09]	1.06 [1.03–1.08]	*0.99 [0.95–1.02]*	*1.01 [0.99–1.03]*	*1.00 [0.97–1.03]*
University or more	*1.00 [0.93–1.07]*	1.12 [1.06–1.18]	1.18 [1.14–1.22]	1.17 [1.13–1.21]	0.93 [0.89–0.97]	0.94 [0.91–0.96]	*1.00 [0.96–1.03]*
**Civil status**							
Unpartnered	0.90 [0.85–0.95]	0.95 [0.91–0.99]	1.23 [1.20–1.26]	1.27 [1.24–1.30]	1.15 [1.11–1.20]	1.20 [1.18–1.22]	1.13 [1.10–1.15]
**Income**							
Q2	*0.99 [0.92–1.08]*	*0.97 [0.91–1.04]*	1.06 [1.02–1.09]	1.07 [1.03–1.10]	*0.99 [0.95–1.04]*	*1.01 [0.98–1.04]*	*1.00 [0.96–1.03]*
Q3	1.11 [1.02–1.20]	*1.04 [0.97–1.11]*	1.05 [1.01–1.09]	1.05 [1.01–1.09]	*1.009 [0.96–1.06]*	0.96 [0.93–0.89]	*1.01[0.97–1.05]*
Q4	1.12 [1.03–1.21]	*1.02 [0.95–1.09]*	*1.00 [0.96–1.03]*	*0.99 [0.95–1.02]*	0.91 [0.86–0.96]	0.87 [0.84–0.90]	*1.03 [0.99–1.07]*
Q5	1.09 [1.00–1.19]	1.10 [1.02–1.18]	*1.00 [0.96–1.04]*	*0.99 [0.95–1.03]*	0.85 [0.80–0.90]	0.79 [0.77–0.82]	*0.98 [0.94–1.02]*
**Polypharmacy status**							
Polypharmacy	2.46 [2.28–2.67]	3.47 [3.23–3.74]	3.59 [3.46–3.72]	3.44 [3.32–3.57]	3.70 [3.48–3.95]	0.53 [0.52–0.54]	1.28 [1.24–1.32]
Excessive polypharmacy	3.80 [3.51–4.12]	6.83 [6.35–7.37]	10.57 [10.18–10.97]	9.91 [9.55–10.29]	9.54 [8.96–10.16]	0.56 [0.55–0.58]	1.92 [1.86–1.98]

AP/OAC, antiplatelets/anticoagulants; CI, confidence interval; CNS, central nervous system; CV, cardiovascular system; FRIDs, Fall Risk Inducing Drugs; MSK, musculoskeletal system; OR, odds ratio; PIM, potentially inappropriate prescribed medications; PPO, potential prescribing omissions; PIM&PPO, having at least one PIM, and one PPO; Q, income quintile. The reference groups are: the complement of the prevalent PIM, and PPO, categories, age range [65–74] years, female sex, elementary education, having a partner, lowest income quintile (Q1), and no polypharmacy. The numbers in Italic suggest that the values were statistically nonsignificant *p* > 0.05.

Male sex was associated with lower odds of experiencing PIM and PIM&PPO. Similar trends were observed for CNS, FRIDs and Analgesics PIM, and MSK PPO. Hower, male sex demonstrated higher odds of CV, AP/OAC PIM, PPO and CV PPO. Older age groups were associated with higher odds of having at least one PIM, PPO and PIM&PPO, along with prevalent PIM/PPO categories, except for AP/OAC and Analgesics PIM. Unpartnered individuals had significantly higher odds of PIM/PPO categories, except for CV and AP/OAC PIM. A higher income was associated with lower odds of PPO, PIM&PPO, Analgesics PIM and CV PPO. Only those in the highest quintile (Q5) had higher odds of AP/OAC PIM compared to those in the lowest quintile (Q1). On the contrary, higher income was associated with higher odds of CV, CNS and FRIDs PIM. No significant association was found between income, overall and CV PIM and MSK PPO. Higher education was associated with higher odds of PIM and PIM&PPO, and with lower odds of PPO and Analgesics PIM. However, no association was found between higher education and MSK PPO, whereas an association was observed with CV PPO.

Additionally, potential associated factors of START criterion A1: *OAC PPO* (Additional file A3: Supplementary Table S2) were explored. Polypharmacy, male sex, and higher income were associated with lower odds of OAC PPO, while older age and being unpartnered were linked to higher odds. However, education was not a statistically significant associated factor for OAC PPO.

### PIM, PPO and prevalent categories associated with adverse health outcomes

**FIGURE 2:**
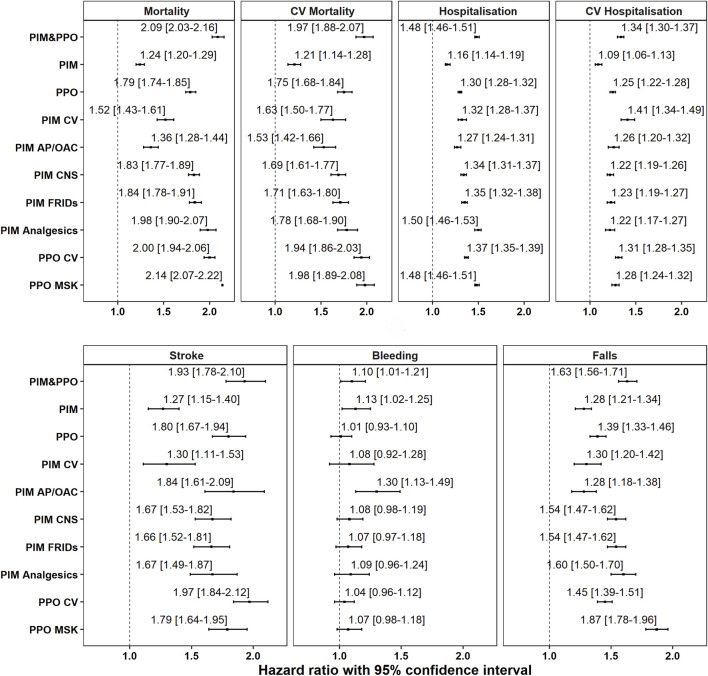
Association between PIM/PPO categories and adverse health outcomes. Estimates are derived from adjusted Cox proportional regression analyses conducted over a 2-year follow-up period. *HR = hazard ratio, CV= cardiovascular, AP/OAC = antiplatelets/anticoagulants, CNS = central nervous system, FRIDs = Fall Risk Increasing Drugs, MSK = musculoskeletal.* Models are adjusted by sex, age, civil status, education, income and polypharmacy.

Multimorbid older adults with AF who had either a PIM or both a PIM and PPO (PIM&PPO) had a higher estimated conditional hazard for all adverse health outcomes compared to those whose medications were all appropriately prescribed. Specifically, among the PIM categories, only PIM of AP/OAC demonstrated a 30% higher estimated conditional hazard for bleeding. Both PPO and prevalent PPO categories had significantly higher hazards for all adverse health outcomes, excluding bleeding, compared to those with all medications appropriately prescribed (Figure 2).

Among older adults with OAC PPO (*START criterion A1*) the risk of stroke, all-cause and CV mortality was found to be twice as high (Additional file A3: Supplementary Table S3). Additionally, after multivariable adjustment, individuals with OAC PPO had a 40% higher risk of experiencing injurious falls and hospitalisation. Finally, no statistically significant association with bleeding was observed compared to those with appropriately prescribed medications.

## Discussion

The present national register-based cohort study comprehensively analysed the prevalence of overall PIP among multimorbid older Swedish adults (≥65 years) with AF. Additionally, the study assessed potential determinants of PIP, prevalent reduced STOPP(PIM)/START(PPO) categories, and their association with adverse health outcomes over a 2-year period.

Our principal findings are: (i) In the total population 39.2% of the participants had at least one PIM and 58.6% at least one PPO, while 24.7% exhibited both a PIM and PPO; (ii) Demographic variables and polypharmacy were identified as positive associated factors of PIM and PPO; (iii) PIM resulted in a higher risk of all adverse health outcomes, while PPO was associated with all outcomes except for bleeding.

### Prevalence of PIM and PPO

In the current literature, the prevalence of PIP varies significantly within the non-AF-specific older population, with PIM ranging from 36.7% to 88.3% and PPO from 29.9% to 85.0% (Tian et al., 2023; Brunetti et al., 2019; Mekonnen et al., 2021; Bare et al., 2022; Abukhalil et al., 2022; Anrys et al., 2018; Brkic et al., 2022). A systematic review assessing the prevalence of PIP in multimorbid and polymedicated older adults with AF highlighted a predominant focus on PIP linked to OAC (Amrouch et al., 2024). The review reported a pooled prevalence of 35.2% for PIP related to OAC. However, considerable variation existed among the included studies, with PIMs of OAC ranging from 8.9% to 56.5%, and PPO ranging from 13.8% to 39.3%. In our study, we observed PIM and PPO of OAC in 5.0% and 34.3% of the population, respectively. Limited knowledge of anticoagulation management among clinicians and patients, and clinicians’ concerns regarding perceived bleeding risks might contribute to the significant omission of appropriate antithrombotic treatment (Andrade et al., 2016; Ding et al., 2017; Osasu et al., 2021; Swed et al., 2024).

Currently, only one other study has assessed prevalence of PIP in older adults with AF, using STOPP/STARTv2 as a medication review tool. Guo et al., 2022 (Guo et al., 2022) reported a higher prevalence of PIM (43.6%) and PPO (71.6%) in their cross-sectional survey on 500 randomly selected patients (≥65 years) of the China Atrial Fibrillation Registry Study, a hospital-based register that enrolled patients from 2011 to 2017. The manual application of all STOPP/STARTv2 criteria and the inclusion of traditional Chinese medicine compounds, which were the most prevalent PIM, may have contributed to the observed higher prevalence. In line with our findings, AP/OAC, ACEIs/ARBs, β-blockers, and statins were the most common PPO (Guo et al., 2022).

The prevalent PPO involving CV medications highlights the insufficient cardiovascular risk prevention (e.g., statins) and ABC-concordant AF management (e.g., β-blockers). Similarly, the high prevalence of PPO related to vitamin D, calcium and bisphosphonates might suggest a disregard for the prevention of osteoporotic fractures (Mekonnen et al., 2021; Bare et al., 2022; Solomon et al., 2003; Steinman et al., 2006; D et al., 2018). However, the over-the-counter availability of calcium and vitamin D, not captured in this study, might also partially explain such high undertreatment. Consistent with other studies (non-AF-specific), CNS and FRIDs PIM were a prevalent phenomenon in older adults with AF and should be one of the key targets for deprescribing (Tommelein et al., 2015; Mekonnen et al., 2021; Bare et al., 2022; D et al., 2018; San-José et al., 2015; Wang et al., 2016).

These prescribing patterns and the prevalence of PIM and PPO emphasise the importance of comprehensive medication reviews, rather than focusing solely on the appropriateness of OAC, in this complex population.

### Factors associated with PIM, PPO and prevalent categories

In this study, patients with polypharmacy, a recognised risk factor for PIM (Tommelein et al., 2015; Tian et al., 2023), showed lower odds of PPO compared to subjects without polypharmacy. The association patterns between polypharmacy and PPO differ across studies (Guo et al., 2022; Steinman et al., 2006; San-José et al., 2015; Blanco-Reina et al., 2015). This might suggest that the number of medications may not reliably predict PPO. While polypharmacy demonstrated a similar trend in CV PPO as for overall PPO, a contrasting relationship was identified concerning MSK PPO. This potentially indicates that clinicians may prioritise prescribing preventive CV medications but are less likely to initiate osteoporosis treatment (vitamin D and calcium) in the more clinically complex individuals. This suggests a complex and multifaceted interplay between polypharmacy, PIM, and PPO.

In older age groups and among unpartnered individuals, the odds of having PIM, PPO, or PIM&PPO rather than appropriately prescribed medications, was significantly higher compared to the reference group. While several studies, in older populations not specific to AF support these findings, others have reported conflicting or nonsignificant results (Tommelein et al., 2015; Tian et al., 2023; Guo et al., 2022; Bare et al., 2023; Bongue et al., 2009). Functional decline and frailty, which increase with advancing age (Hajek and Konig, 2016; Hajek et al., 2016), have both been associated with PIM and PPO (Zuleta et al., 2024; Tommelein et al., 2017). Additionally, unpartnered older adults, especially those living alone, have been associated with greater functional decline, increased hospitalisation, and higher mortality rates (Hajek and Konig, 2016; Pimouguet et al., 2017; Holt-Lunstad et al., 2015).

Male sex was associated with lower odds of PIM and PIM&PPO, but higher odds of PPO, aligning with previous findings (Bare et al., 2023; Johnell et al., 2009; Morgan et al., 2016; Buzancic et al., 2024). Other studies have reported null associations between sex and PIP (Tommelein et al., 2015; Hill-Taylor et al., 2013). Our study population might show a higher clinical complexity, characterised by multimorbidity and polypharmacy. Additionally, our focus is on sex differences, but gender-related sociocultural factors might also play a part in the observed PIM and PPO patterns (Rochon et al., 2021).

Higher income was associated with lower odds of PPO and PIM&PPO, while no statistically significant association was found with PIM. In contrast, higher educational attainment was statistically significantly associated with increased odds of PIM and PIM&PPO, and lower odds of PPO. Interestingly, Hwang et al., 2022 used, in addition, a cumulative socioeconomic status score based on education, income and area of deprivation index, which revealed a lower socioeconomic status score to be significantly associated with higher odds of PIM (Hwang et al., 2022). This suggests that a cumulative socioeconomic status score may better explain the association with PIM. Additionally, individuals with higher education may tend to seek second opinions and consult multiple healthcare providers, contributing to fragmentation of care (Tam et al., 2005; Okamoto et al., 2015). This fragmentation, in turn, has been associated with higher rates of PIM and mortality (Prior et al., 2023). Addressing fragmented care through the improvement of integrated healthcare systems could serve as a strategy to reduce instances of PIM.

These findings clearly underscore the importance of conducting personalised and targeted medication reviews, with careful consideration given the clinical, but also demographic factors associated with PIM and PPO outcomes.

### Association of PIM and PPO with adverse health outcomes

The association between PIM and adverse health outcomes in older adults, regardless of AF, has shown conflicting results, with some studies showing an association and others not (Mekonnen et al., 2021; Mekonnen et al., 2022; Wauters et al., 2016; Veldhuis et al., 2023). Specifically, AP/OAC PIM was associated with a 30% higher risk of bleeding, corroborating clinicians’ concerns on bleeding complications with antithrombotic treatment (Andrade et al., 2016; Ding et al., 2017; Osasu et al., 2021; Swed et al., 2024). Similarly, individuals with PPO demonstrated a higher hazard for all adverse health outcomes except for bleeding, a trend documented in prior research (Mekonnen et al., 2021; Wauters et al., 2016; Cardwell et al., 2020).

Moreover, individuals with PPO&PIM had twice the risk of mortality compared to those with appropriate prescribing, highlighting the importance of assessing both PIM and PPO. Previous studies have also linked CNS and FRIDs PIM, along with MSK and CV PPO, to drug-related hospitalisations, an aspect not examined in our study but nonetheless relevant (Mekonnen et al., 2021).

Furthermore, PPO involving MSK system medications was associated with an 87% higher estimated conditional hazard for injurious falls, indicating the potential role of appropriate prescribing of vitamin D, calcium, and antiresorptive medications in fall prevention (LeBoff et al., 2022). Additionally, PPO of CV preventive medications, particularly antithrombotic therapy for stroke prevention, was associated with mortality, stroke, hospitalisation, and injurious falls. Thus, it is essential to deprescribe CNS medications and ensure appropriate prescribing of preventive MSK medications to mitigate fall risks, as well as OAC treatment to prevent strokes. Thomas et al., 2023 also demonstrated a higher risk of mortality for those with PPO, especially CV PPO, and reported a significant decrease in mortality when these omissions were corrected, suggesting that addressing PPO could be beneficial (Thomas et al., 2023).

These findings highlight the importance of prioritising deprescribing interventions and optimising preventive prescribing practices to mitigate the risk of adverse health outcomes.

### Clinical implications

The identified prescribing patterns enable a focused medication review targeting prevalent PIM and PPO. Moreover, this study provides comprehensive insights into the demographic and clinical characteristics of individuals susceptible to PIM and PPO, alongside with the associated adverse health outcomes. It is essential to acknowledge that these PIM and PPO may not always be inappropriate in specific clinical contexts and settings (Parodi Lopez et al., 2022). The decision to initiate or withhold medication in multimorbid older adults is complex and often determined on a case-by-case basis, considering individual patient factors. Therefore, a qualitative assessment of the reasons behind the PIM or PPO of medications is crucial for gaining a deeper understanding of the clinician’s prescribing decisions.

Medication review and judicious deprescribing in older inpatients can reduce PIM and reduce hospital readmission rates (Carollo et al., 2024). Several strategies have been developed and evaluated, highlighting the importance of the regular performance of medication reviews, continuous communication between healthcare professionals, a multidisciplinary team approach, and comprehensive documentation. These factors are key contributors to the effectiveness of interventions aimed at improving prescribing practices and patient related outcomes (Carollo et al., 2024).

Given the complex health profile of multimorbid and polymedicated older adults with AF, there is a recognised imperative for a holistic and patient-tailored healthcare provision. The AFFIRMO framework aims to improve the therapeutic management, according to the ABC (The Atrial fibrillation Better Care pathway) integrated care approach for this study population (Lip, 2017). These findings may contribute to shaping the AFFIRMO framework, especially in terms of risk stratification.

### Limitations

A limitation of this study is that not all STOPP/STARTv2 categories could be assessed due to restrictions of the registries. Assumptions may have led to an over- or underestimation of PIP prevalence. Moreover, while multicollinearity prevented the inclusion of the number of diseases in the association analyses, the multimorbidity profile of these patients may also contribute to understanding PIP and prescribing patterns, emphasising the need for assessment in future studies.

Although the third version of STOPP/START is available, the second version was used in this study due to the lack of a technical translation for the third version. The second version may be considered outdated. For instance, current European Society of Cardiology guidelines advise against monotherapy of antiplatelets for stroke prevention (criterion STARTA2) in adults with AF (Hindricks et al., 2021). Despite this potential limitation, the second version was preferred because Huibers et al., 2019 provide an operationalisation of the reduced STOPP/STARTv2, developed through a multidisciplinary consensus procedure (Huibers et al., 2019). This ensures replicability and improves comparability.

Finally, we studied a predominantly white European population in Sweden, and further investigation is warranted to generalise our findings to other ethnic groups or to populations in low- and middle-income countries, given recognised ethnic differences in AF and AF-related complications (Kang et al., 2024).

## Conclusion

The present study reports a high prevalence of PIP in multimorbid older adults with AF. Additionally, the association analyses highlight a nuanced relationship between prescribing patterns, patient characteristics, and adverse health outcomes. These findings emphasise the importance of tailored interventions to optimise medication management in this patient population.

## Data Availability

The datasets presented in this article are not readily available because, according to the Swedish Ethical Review Act, the General Data Protection Regulation, the Public Access to Information and Secrecy Act, registry data can only be made available after legal review for researchers who meet the criteria for access to this type of confidential data. Requests to access the datasets should be directed to socialstyrelsen: socialstyrelsen@socialstyrelsen.se.

## Footnotes

1
https://www.socialstyrelsen.se/en/statistics-and-data/registers/

3
https://www.socialstyrelsen.se/en/statistics-and-data/registers/national-patient-register/

4
https://www.socialstyrelsen.se/en/statistics-and-data/registers/national-prescribed-drug-register/

2
https://rut.registerforskning.se/metadatakatalog/register/40a71c3f-ad03-4677-84bd-7850db9a8f3a/
