# Supplementary material for: Potentially inappropriate prescribing in polymedicated older adults with atrial fibrillation and multimorbidity: a Swedish national register-based cohort study
Source: Front Pharmacol. 2024 Sep 10;15:1476464. doi: 10.3389/fphar.2024.1476464 (PMC11420530; doi:10.3389/fphar.2024.1476464)
Supplement: Supplementary file 1 [file DataSheet2.PDF]

````
```{r setup, include=FALSE}
knitr::opts_chunk$set(echo = TRUE)
```

# set directory
```{r}
setwd(dir="")
```

# install packages
```{r}
library(foreign)
library(dplyr)
library(MASS)
library(ZIM)
library(lattice)
require(pscl)
library(lmtest)
library(pander)
library(ggeffects)
library(patchwork)
library(olsrr)
library(mice)
library(haven)
library(memisc)
```

# Reading in file
```{r}
```

# Drugs and Diseases need to be in long format.
# Coding the STOPP criteria:
## Start writing the code for Drugs related to the cardiovascular system.

### STOPPB1 Stop digoxin for heart failure with normal systolic ventricular function.
```{r}
SNPR <- mutate (SNPR, STOPPB1 = if_else(SNPR$ATC == "C01AA05" & substr(SNPR$Diseases,1,4)=="I503", 1, 0))
SNPR <- SNPR %>% group_by(lopnr) %>% mutate(STOPPB1max = max(STOPPB1)) %>% ungroup()
```

### STOPPB3 Stop beta-blocker in combination with verapamil or diltiazem (risk of heart block).
```{r}
SNPR <- mutate (SNPR, VERAP_DILTIAZ = if_else((SNPR$ATC == "C08DA01" | SNPR$ATC=="C08DA51" | SNPR$ATC=="C09BB10" | SNPR$ATC
=="C08DB01"), 1, 0))
SNPR <- SNPR %>% group_by(lopnr) %>% mutate(VERAP_DILTIAZmax = max(VERAP_DILTIAZ)) %>% ungroup()

SNPR <- mutate (SNPR, STOPPB3 = if_else (substr(SNPR$ATC,1,3) == "C07" & SNPR$VERAP_DILTIAZmax==1, 1, 0))
SNPR <- SNPR %>% group_by(lopnr) %>% mutate(STOPPB3max = max(STOPPB3)) %>% ungroup()
```

### STOPPB4 Stop beta-blocker with bradycardia (<50/min), type II heart block or complete heart block (risk of complete heart block,
asystole).
```{r}
SNPR <- mutate (SNPR, STOPPB4 = if_else(substr(SNPR$ATC,1,3)=="C07" & (substr(SNPR$Diseases,1,4)=="I441"  |
substr(SNPR$Diseases,1,4)=="I442" | substr(SNPR$Diseases,1,4)=="I443" | substr(SNPR$Diseases,1,4)=="I455" |
substr(SNPR$Diseases,1,4)=="I459" | substr(SNPR$Diseases,1,4)=="Q246" | substr(SNPR$Diseases,1,4)=="I441"), 1, 0))
SNPR <- SNPR %>% group_by(lopnr) %>% mutate(STOPPB4max = max(STOPPB4)) %>% ungroup()
```

### STOPPB7 Stop Loop diuretic for dependent ankle oedema without clinical, biochemical evidence or radiological evidence of heart
failure, liver failure, nephrotic syndrome or renal failure
```{r}
SNPR <- mutate (SNPR, failure = if_else(substr(SNPR$Diseases,1,3) == "I50" | substr(SNPR$Diseases,1,4) == "I110" |
substr(SNPR$Diseases,1,4) == "I130" | substr(SNPR$Diseases,1,4) == "I132" |substr(SNPR$Diseases,1,4) == "K704" |
substr(SNPR$Diseases,1,3) == "K72" | substr(SNPR$Diseases,1,3) == "K73" |  substr(SNPR$Diseases,1,3) == "N03" |
substr(SNPR$Diseases,1,3) == "N04" | substr(SNPR$Diseases,1,3) == "N05" | substr(SNPR$Diseases,1,3) == "N17" |
substr(SNPR$Diseases,1,3) == "N18" | substr(SNPR$Diseases,1,3) == "N19",1,0))
SNPR <- SNPR %>% group_by(lopnr) %>% mutate(failuremax = max(failure))  %>% ungroup()

SNPR <- mutate(SNPR, STOPPB7 = if_else((substr(SNPR$ATC,1,4)=="C03C" | substr(SNPR$ATC,1,5)=="C03EB") &
substr(SNPR$Diseases,1,4)=="R600" & SNPR$failuremax == 0,1,0))
SNPR <- SNPR %>% group_by(lopnr) %>% mutate(STOPPB7max = max(STOPPB7))  %>% ungroup()
```

### STOPPB8 Stop thiazide diuretic with current significant hypokalaemia (i.e. serum K+ < 3.0 mmol/l), hyponatraemia (i.e. serum Na+
< 130 mmol/l) hypercalcaemia (i.e. corrected serum calcium > 2.65 mmol/l) or with a history of gout (hypokalaemia, hyponatraemia,
hypercalcaemia and gout can be precipitated by thiazide diuretic) (i.e. corrected serum calcium > 2.65 mmol/l).
```{r}
SNPR <- mutate (SNPR, STOPPB8= if_else ((substr(SNPR$ATC,1,4)=="C03A" | substr(SNPR$ATC,1,5)=="C03BA" | SNPR$ATC=="C03EA01" |
SNPR$ATC=="C03EA02" | SNPR$ATC=="C03EA13" | SNPR$ATC=="C03EA07" | SNPR$ATC=="C09XA52" | SNPR$ATC=="C09XA54" |
substr(SNPR$ATC,1,4)=="C07B" | substr(SNPR$ATC,1,4)=="C07D" | SNPR$ATC=="C09DX01" | SNPR$ATC=="C09DX03" ) &
(substr(SNPR$Diseases,1,4)=="E876" | substr(SNPR$Diseases,1,4)=="E871" | substr(SNPR$Diseases,1,3)=="M10"),1,0))
SNPR <- SNPR %>% group_by(lopnr) %>% mutate(STOPPB8max = max(STOPPB8)) %>% ungroup()
```

### STOPPB9 Stop Loop diuretic for treatment of hypertension with concurrent urinary incontinence (may exacerbate incontinence).
```{r}
SNPR <- mutate(SNPR, UrinaryIncontinence= if_else (substr(SNPR$Diseases,1,4)=="N393" | substr(SNPR$Diseases,1,4)=="N394" |
substr(SNPR$Diseases,1,3)=="R32",1,0))
SNPR <- SNPR %>% group_by(lopnr) %>% mutate(UrinaryIncontinencemax = max(UrinaryIncontinence)) %>% ungroup()

SNPR <- mutate (SNPR, Heartfailure= if_else (substr(SNPR$Diseases,1,3)=="I50" | substr(SNPR$Diseases,1,4)=="I110" |
substr(SNPR$Diseases,1,4)=="I130" | substr(SNPR$Diseases,1,4)=="I132",1,0))
SNPR <- SNPR %>% group_by(lopnr) %>% mutate(Heartfailuremax = max(Heartfailure)) %>% ungroup()

SNPR <- mutate(SNPR, STOPPB9= if_else((substr(SNPR$ATC,1,4)=="C03C" | substr(SNPR$ATC,1,5)=="C03EB" ) &
(substr(SNPR$Diseases,1,3)=="I10" | substr(SNPR$Diseases,1,3)=="I15")  & SNPR$UrinaryIncontinencemax==1 &
SNPR$Heartfailuremax==0,1,0))
SNPR <- SNPR %>% group_by(lopnr) %>% mutate(STOPPB9max = max(STOPPB9)) %>% ungroup()
```
````

STOPPB11 Stop ACE inhibitors or Angiotensin Receptor Blockers in patients with hyperkalaemia.
```{r}
SNPR <- mutate(SNPR, STOPPB11= if_else(substr(SNPR$ATC,1,3)=="C09" & substr(SNPR$Diseases,1,4)=="E875",1,0))
SNPR <- SNPR %>% group_by(lopnr) %>% mutate(STOPPB11max = max(STOPPB11)) %>% ungroup()
```

STOPPB13 Stop phosphodiesterase type-5 inhibitors (e.g. sildenafil, tadalafil, vardenafil) in severe heart failure characterized by hypotension i.e. systolic BP < 90 mmHg, or concurrent nitrate therapy for angina (risk of cardiovascular collapse).
####STOPPB13_2 Phosphodiesterase type 5 inhibitors (sildenafil, tadalafil, vardenafil) AND heart failure AND nitrate therapy (C01DA) for Angina Pectoris I20.
```{r}
SNPR <- mutate(SNPR, Nitrate= if_else(substr(SNPR$ATC,1,5)=="C01DA",1,0))
SNPR <- SNPR %>% group_by(lopnr) %>% mutate(Nitratemax = max(Nitrate)) %>% ungroup()

SNPR <- mutate(SNPR, STOPPB13_2 = if_else((SNPR$ATC=="G04BE10" | SNPR$ATC=="G04BE03" | SNPR$ATC=="G04BE08" | SNPR$ATC=="G04BE09") & SNPR$Nitratemax==1 & substr(SNPR$Diseases,1,3)=="I20",1,0))
SNPR <- SNPR %>% group_by(lopnr) %>% mutate(STOPPB13_2max = max(STOPPB13_2)) %>% ungroup()
```

Total STOPPB_Cardiovascular
```{r}
SNPR <- mutate (SNPR, STOPPB_Cardiovascular = STOPPB1max + STOPPB3max + STOPPB4max + STOPPB7max + STOPPB8max + STOPPB9max + STOPPB11max + STOPPB13_2max)
```

STOPPC CRITERIA ANTIPLATELET / ANTICOAGULANT
STOPPC2 Stop aspirin with a past history of peptic ulcer disease without concomitant PPI (risk of recurrent peptic ulcer).
```{r}
SNPR <- mutate(SNPR, PPI= if_else(substr(SNPR$ATC,1,5)=="A02BC",1,0))
SNPR <- SNPR %>% group_by(lopnr) %>% mutate(PPImax = max(PPI)) %>% ungroup()
```

```{r}
SNPR <- mutate(SNPR, STOPPC2= if_else((SNPR$ATC=="B01AC06" | SNPR$ATC=="B01AC08" | SNPR$ATC=="N02BA01" | SNPR$ATC=="M01BA03" | SNPR$ATC=="C10BX01" | SNPR$ATC=="C10BX02" | SNPR$ATC=="C10BX04" | SNPR$ATC=="C10BX05" | SNPR$ATC=="C10BX06" | SNPR$ATC=="C10BX08" | SNPR$ATC=="N02BA15" | SNPR$ATC=="N02BA51" | SNPR$ATC=="N02BA65" | SNPR$ATC=="N02BA71") & ( substr(SNPR$Diseases,1,4)=="K221" | substr(SNPR$Diseases,1,3)=="K25" |  substr(SNPR$Diseases,1,3)=="K26" |  substr(SNPR$Diseases,1,3)=="K27" | substr(SNPR$Diseases,1,3)=="K28") & SNPR$PPImax == 0,1,0))
SNPR <- SNPR %>% group_by(lopnr) %>% mutate(STOPPC2max = max(STOPPC2)) %>% ungroup()
```

STOPPC3 Stop Aspirin, clopidogrel, dipyridamole, vitamin K antagonists, direct thrombin inhibitors or factor Xa inhibitors with concurrent significant bleeding risk, i.e. uncontrolled severe hypertension, bleeding diathesis, recent non-trivial spontaneous bleeding) (high risk of bleeding).
```{r}
SNPR <- mutate(SNPR, STOPPC3= if_else((substr(SNPR$ATC,1,5)=="B01AA"  | substr(SNPR$ATC,1,5) == "B01AC" | substr(SNPR$ATC,1,5) == "B01AE" | substr(SNPR$ATC,1,5)=="B01AF")  & (substr(SNPR$Diseases,1,3)=="D66" | substr(SNPR$Diseases,1,3)=="D67" | substr(SNPR$Diseases,1,4)=="D680" | substr(SNPR$Diseases,1,4)=="D693" | substr(SNPR$Diseases,1,3)=="I60" | substr(SNPR$Diseases,1,3)=="I61" | substr(SNPR$Diseases,1,3)=="I62" | substr(SNPR$Diseases,1,3)=="I64") , 1 ,0))
SNPR <- SNPR %>% group_by(lopnr) %>% mutate(STOPPC3max = max(STOPPC3)) %>% ungroup()
```

STOPPC4 Stop Aspirin plus clopidogrel as secondary stroke prevention, unless the patient has a coronary stent(s) inserted in the previous 12 months or concurrent acute coronary syndrome or has a high grade symptomatic carotid arterial stenosis
```{r}
SNPR <- mutate(SNPR, Aspirin= if_else(SNPR$ATC=="B01AC06" | SNPR$ATC=="B01AC08" | SNPR$ATC=="B01AC56" | SNPR$ATC == "B01AC30",1,0))
SNPR <- SNPR %>% group_by(lopnr) %>% mutate(Aspirinmax = max(Aspirin)) %>% ungroup()
```

```{r}
SNPR <- mutate(SNPR, Coronary= if_else(substr(SNPR$Diseases,1,4)=="I200" |  substr(SNPR$Diseases,1,4)=="I210",1,0))
SNPR <- SNPR %>% group_by(lopnr) %>% mutate(Coronarymax = max(Coronary)) %>% ungroup()
```

```{r}
SNPR <- mutate(SNPR, STOPPC4= if_else(SNPR$ATC =="B01AC04" & Aspirinmax == 1 & Coronarymax == 0 & (substr(SNPR$Diseases,1,3)=="I63" | substr(SNPR$Diseases,1,3)=="I64" | substr(SNPR$Diseases,1,3)=="G45"), 1 ,0))
SNPR <- SNPR %>% group_by(lopnr) %>% mutate(STOPPC4max = max(STOPPC4)) %>% ungroup()
```

STOPPC5 Stop aspirin in combination with vitamin K antagonist, direct thrombin inhibitor or factor Xa inhibitors in patients with chronic atrial fibrillation (no added benefit from aspirin).
```{r}
SNPR <- mutate(SNPR, Anticoagulantia= if_else((substr(SNPR$ATC,1,5)=="B01AA" | substr(SNPR$ATC,1,5) == "B01AE" | substr(SNPR$ATC,1,5)=="B01AF"),1,0))
SNPR <- SNPR %>% group_by(lopnr) %>% mutate(Anticoagulantiamax = max(Anticoagulantia)) %>% ungroup()
```

```{r}
SNPR <- mutate(SNPR, STOPPC5=if_else((SNPR$ATC=="B01AC06" | SNPR$ATC=="B01AC08" | SNPR$ATC=="B01AC56") & SNPR$Anticoagulantiamax==1 & substr(SNPR$Diseases,1,3)=="I48",1,0))
SNPR <- SNPR %>% group_by(lopnr) %>% mutate(STOPPC5max = max(STOPPC5)) %>% ungroup()
```

STOPPC7 Stop Ticlopidine in any circumstances (clopidogrel and prasugrel have similar efficacy, stronger evidence and fewer side-effects).
```{r}
SNPR <- mutate(SNPR, STOPPC7= if_else(SNPR$ATC=="B01AC05",1,0))
SNPR <- SNPR %>% group_by(lopnr) %>% mutate(STOPPC7max = max(STOPPC7)) %>% ungroup()
```

STOPPC10 Stop NSAID and vitamin K antagonist, direct thrombin inhibitor or factor Xa inhibitors in combination (risk of major gastrointestinal bleeding).
```{r}
SNPR <- mutate(SNPR,STOPPC10 = if_else((substr(SNPR$ATC,1,4)=="M01A" | substr(SNPR$ATC,1,5)=="N02BA") & SNPR$Anticoagulantiamax==1,1,0))
SNPR <- SNPR %>% group_by(lopnr) %>% mutate(STOPPC10max = max(STOPPC10)) %>% ungroup()
```

STOPPC11 Stop NSAID with concurrent antiplatelet agent(s) without PPI prophylaxis (increased risk of peptic ulcer disease)
```{r}
SNPR <- mutate(SNPR,Antiplatelet= if_else(substr(SNPR$ATC,1,5)=="B01AC" & SNPR$ATC != "B01AC56",1,0))
SNPR <- SNPR %>% group_by(lopnr) %>% mutate(Antiplateletmax = max(Antiplatelet)) %>% ungroup()
```

````
SNPR <- mutate(SNPR,STOPPC11= if_else((substr(SNPR$ATC,1,4)=="M01A" | substr(SNPR$ATC,1,5)=="N02BA") & SNPR$Antiplateletmax==1 &
SNPR$PPImax == 0,1,0))
SNPR <- SNPR %>% group_by(lopnr) %>% mutate(STOPPC11max = max(STOPPC11)) %>% ungroup()
```

## STOPPC Antiplatelet Anticoagulant total
```{r}
SNPR <- mutate (SNPR,STOPPC_AntiplateletAnticoagulent= STOPPC2max + STOPPC3max + STOPPC4max + STOPPC5max + STOPPC7max + STOPPC10max
+ STOPPC11max)
```

## STOPP D CNS
### STOPPD1 Stop TriCyclic Antidepressants (TCAs) with dementia, narrow angle glaucoma, cardiac conduction abnormalities,
prostatism, or prior history of urinary retention
```{r}
SNPR <- mutate(SNPR,STOPPD1= if_else((substr(SNPR$ATC,1,5)== "N06AA" | SNPR$ATC=="N06CA01" | SNPR$ATC=="N06CA02") &
                                        (substr(SNPR$Diseases,1,3)=="F00" | substr(SNPR$Diseases,1,3)=="F01" |
substr(SNPR$Diseases,1,3)=="F02"
                                         | substr(SNPR$Diseases,1,3)=="F03" | substr(SNPR$Diseases,1,4)=="H402" |
substr(SNPR$Diseases,1,3)=="I44"
                                         | substr(SNPR$Diseases,1,3)=="I45" | substr(SNPR$Diseases,1,3)=="G30" |
substr(SNPR$Diseases,1,4)=="G310"
                                         | substr(SNPR$Diseases,1,4)=="G311" | substr(SNPR$Diseases,1,4)=="G318" |
substr(SNPR$Diseases,1,3)=="N40"
                                         | substr(SNPR$Diseases,1,3)=="R33"),1,0))

SNPR <- SNPR %>% group_by(lopnr) %>% mutate(STOPPD1max = max(STOPPD1)) %>% ungroup()

```

###STOPPD3 Stop Neuroleptics with moderate-marked antimuscarinic/anticholinergic effects (chlorpromazine, clozapine, flupenthixol,
fluphenzine, pipothiazine, promazine, zuclopenthixol) with a history of prostatism or previous urinary retention
```{r}
SNPR <- mutate(SNPR,STOPPD3= if_else((SNPR$ATC == "N05AA01" | SNPR$ATC == "N05AH02" | SNPR$ATC == "N05AF01" | SNPR$ATC == "N05AB02"
| SNPR$ATC == "N05AC04"
                                       | SNPR$ATC == "N05AA03" | SNPR$ATC == "N05AF05") & (substr(SNPR$Diseases,1,3)=="N40"
                                       | substr(SNPR$Diseases,1,3) == "R33"),1,0))

SNPR <- SNPR %>% group_by(lopnr) %>% mutate(STOPPD3max = max(STOPPD3)) %>% ungroup()
```

###STOPPD4 Stop Selective serotonin re-uptake inhibitors (SSRI’s) with current or recent significant hyponatraemia i.e. serum Na+ <
130 mmol/l
```{r}
SNPR <- mutate(SNPR,STOPPD4= if_else((substr(SNPR$ATC,1,5)== "N06AB" | SNPR$ATC == "N06CA03") &
(substr(SNPR$Diseases,1,4)=="E871"),1,0))
SNPR <- SNPR %>% group_by(lopnr) %>% mutate(STOPPD4max = max(STOPPD4)) %>% ungroup()
```

### STOPPD5 Stop benzodiazepines for> 4 weeks (no indication for longer treatment; risk of prolonged sedation, confusion, impaired
balance, falls, road traffic accidents; all benzodiazepines should be withdrawn gradually if taken for more than  4  weeks as there
is a risk of causing a benzodiazepine withdrawal syndrome if stopped abruptly).
```{r}
SNPR <- mutate(SNPR,STOPPD5= if_else(substr(SNPR$ATC,1,5)== "N05BA" | substr(SNPR$ATC,1,5)=="N05CD" | substr(SNPR$ATC,1,5)=="N05CF"
| substr(SNPR$ATC,1,5)=="N03AE",1,0))
SNPR <- SNPR %>% group_by(lopnr) %>% mutate(STOPPD5max = max(STOPPD5)) %>% ungroup()
```

### STOPPD6 Stop Antipsychotics (i.e. other than quetiapine or clozapine) in those with parkinsonism or Lewy Body Disease
```{r}
SNPR <- mutate(SNPR,Antipsychotics= if_else(substr(SNPR$ATC,1,5)== "N05AN" |SNPR$ATC == "N05AH02" | SNPR$ATC == "N05AH04",1,0))
SNPR <- SNPR %>% group_by(lopnr) %>% mutate(Antipsychoticsmax = max(Antipsychotics)) %>% ungroup()

SNPR <- mutate(SNPR,STOPPD6= if_else(substr(SNPR$ATC,1,4)== "N05A"  & Antipsychoticsmax == 0 & (substr(SNPR$Diseases,1,3)=="G30" |
substr(SNPR$Diseases,1,3)=="G20"| substr(SNPR$Diseases,1,3)=="G21" | substr(SNPR$Diseases,1,4)=="G231" |
substr(SNPR$Diseases,1,4)=="G232" | substr(SNPR$Diseases,1,4)=="G318" | substr(SNPR$Diseases,1,4)=="G903") ,1,0))
SNPR <- SNPR %>% group_by(lopnr) %>% mutate(STOPPD6max = max(STOPPD6)) %>% ungroup()
```

### STOPPD7 Stop Anticholinergics/antimuscarinics to treat extra-pyramidal side-effects of neuroleptic medications
```{r}
SNPR <- mutate(SNPR,STOPPD7= if_else((SNPR$ATC=="R06AE03" | SNPR$ATC==" R06AE04" |SNPR$ATC=="R06AE05" |
substr(SNPR$ATC,1,5)=="R06AA" | substr(SNPR$ATC,1,5)=="R06AB" | substr(SNPR$ATC,1,5)=="R06AC" | substr(SNPR$ATC,1,5)=="R06AD"  |
SNPR$ATC=="R06AE01" | SNPR$ATC=="R06AE03" | SNPR$ATC=="R06AE04" | SNPR$ATC=="R06AE05" | SNPR$ATC=="R06AE06" | SNPR$ATC=="R06AE51" |
SNPR$ATC=="R06AE53" | SNPR$ATC=="R06AE55" | SNPR$ATC=="R06AX01" | SNPR$ATC=="R06AX02" | SNPR$ATC=="R06AX03" | SNPR$ATC=="R06AX04" |
SNPR$ATC=="R06AX05" | SNPR$ATC=="R06AX08" | SNPR$ATC=="R06AX09" | SNPR$ATC=="R06AX15" | SNPR$ATC=="R06AX16" | SNPR$ATC=="R06AX17" |
SNPR$ATC=="R06AX23" | SNPR$ATC=="R06AX53" | SNPR$ATC=="R06AX58" | SNPR$ATC=="N05BB01" | SNPR$ATC=="N05BB51" | SNPR$ATC=="N07CA02" |
SNPR$ATC=="N07CA52" | SNPR$ATC=="G04BD01" | SNPR$ATC=="G04BD02" | SNPR$ATC=="G04BD03" | SNPR$ATC=="G04BD04" | SNPR$ATC=="G04BD05" |
SNPR$ATC=="G04BD06" | SNPR$ATC=="G04BD07" | SNPR$ATC=="G04BD08" | SNPR$ATC=="G04BD09" | SNPR$ATC=="G04BD10" | SNPR$ATC=="G04BD11" |
substr(SNPR$ATC,1,5)=="N06AA" | substr(SNPR$ATC,1,4)=="A03B" | substr(SNPR$ATC,1,5)=="A03AA" | substr(SNPR$ATC,1,5)=="A03AB" |
SNPR$ATC=="A04AD01" | SNPR$ATC=="M03BX02" | substr(SNPR$ATC,1,4)=="N04A" | SNPR$ATC=="N05AA01" | SNPR$ATC=="N05AH02" |
SNPR$ATC=="N05AB02" | SNPR$ATC=="N05AA02" | SNPR$ATC=="N05AC02" | SNPR$ATC=="N05AA04" | SNPR$ATC=="N05AF04") &
(substr(SNPR$Diseases,1,4)=="G251" | substr(SNPR$Diseases,1,4)=="G254"| substr(SNPR$Diseases,1,5)=="G2561" |
substr(SNPR$Diseases,1,4)=="G257") ,1,0))

SNPR <- SNPR %>% group_by(lopnr) %>% mutate(STOPPD7max = max(STOPPD7)) %>% ungroup()
```

### STOPPD8 Anticholinergics/antimuscarinics in patients with delirium or dementia
```{r}
SNPR <- mutate(SNPR,STOPPD8= if_else((SNPR$ATC=="R06AE03" | SNPR$ATC==" R06AE04" |SNPR$ATC=="R06AE05" |
substr(SNPR$ATC,1,5)=="R06AA" | substr(SNPR$ATC,1,5)=="R06AB" | substr(SNPR$ATC,1,5)=="R06AC" | substr(SNPR$ATC,1,5)=="R06AD"  |
SNPR$ATC=="R06AE01" | SNPR$ATC=="R06AE03" | SNPR$ATC=="R06AE04" | SNPR$ATC=="R06AE05" | SNPR$ATC=="R06AE06" | SNPR$ATC=="R06AE51" |
SNPR$ATC=="R06AE53" | SNPR$ATC=="R06AE55" | SNPR$ATC=="R06AX01" | SNPR$ATC=="R06AX02" | SNPR$ATC=="R06AX03" | SNPR$ATC=="R06AX04" |
SNPR$ATC=="R06AX05" | SNPR$ATC=="R06AX08" | SNPR$ATC=="R06AX09" | SNPR$ATC=="R06AX15" | SNPR$ATC=="R06AX16" | SNPR$ATC=="R06AX17" |
SNPR$ATC=="R06AX23" | SNPR$ATC=="R06AX53" | SNPR$ATC=="R06AX58" | SNPR$ATC=="N05BB01" | SNPR$ATC=="N05BB51" | SNPR$ATC=="N07CA02" |
SNPR$ATC=="N07CA52" | SNPR$ATC=="G04BD01" | SNPR$ATC=="G04BD02" | SNPR$ATC=="G04BD03" | SNPR$ATC=="G04BD04" | SNPR$ATC=="G04BD05" |
SNPR$ATC=="G04BD06" | SNPR$ATC=="G04BD07" | SNPR$ATC=="G04BD08" | SNPR$ATC=="G04BD09" | SNPR$ATC=="G04BD10" | SNPR$ATC=="G04BD11" |
substr(SNPR$ATC,1,5)=="N06AA" | substr(SNPR$ATC,1,4)=="A03B" | substr(SNPR$ATC,1,5)=="A03AA" | substr(SNPR$ATC,1,5)=="A03AB" |
SNPR$ATC=="A04AD01" | SNPR$ATC=="M03BX02" | substr(SNPR$ATC,1,4)=="N04A" | SNPR$ATC=="N05AA01" | SNPR$ATC=="N05AH02" |
SNPR$ATC=="N05AB02" | SNPR$ATC=="N05AA02" | SNPR$ATC=="N05AC02" | SNPR$ATC=="N05AA04" | SNPR$ATC=="N05AF04") &
(substr(SNPR$Diseases,1,3)=="F00" | substr(SNPR$Diseases,1,3)=="F01" | substr(SNPR$Diseases,1,3)=="F02" |
substr(SNPR$Diseases,1,3)=="F03" | substr(SNPR$Diseases,1,3)=="F05" | substr(SNPR$Diseases,1,3)=="G30" |
substr(SNPR$Diseases,1,4)=="G310" | substr(SNPR$Diseases,1,4)=="G311" | substr(SNPR$Diseases,1,4)=="G318") ,1,0))
````

SNPR <- SNPR %>% group_by(lopnr) %>% mutate(STOPPD8max = max(STOPPD8)) %>% ungroup()
```

###STOPPD10 Neuroleptics as hypnotics, unless sleep disorder is due to psychosis or dementia
```{r}
SNPR <- mutate(SNPR, Lithium= if_else(substr(SNPR$ATC,1,5)== "N05AN",1,0))
SNPR <- SNPR %>% group_by(lopnr) %>% mutate(Lithiummax = max(Lithium)) %>% ungroup()

SNPR <- mutate(SNPR, Psych= if_else(substr(SNPR$Diseases,1,3)=="F00" | substr(SNPR$Diseases,1,3)=="F01" |
substr(SNPR$Diseases,1,3)=="F02" | substr(SNPR$Diseases,1,3)=="F03" | substr(SNPR$Diseases,1,3)=="F07" |
substr(SNPR$Diseases,1,3)=="F20" | substr(SNPR$Diseases,1,3)=="F25" | substr(SNPR$Diseases,1,3)=="F29" |
substr(SNPR$Diseases,1,3)=="G30" | substr(SNPR$Diseases,1,4)=="G310" | substr(SNPR$Diseases,1,4)=="G311" |
substr(SNPR$Diseases,1,4)=="G318" ,1,0))
SNPR <- SNPR %>% group_by(lopnr) %>% mutate(Psychmax = max(Psych)) %>% ungroup()

SNPR <- mutate(SNPR,STOPPD10= if_else( substr(SNPR$ATC,1,5)== "N05A" & Lithiummax == 0 & Psychmax == 0 &
(substr(SNPR$Diseases,1,4)=="F510" | substr(SNPR$Diseases,1,4)=="G470") ,1,0))
SNPR <- SNPR %>% group_by(lopnr) %>% mutate(STOPPD10max = max(STOPPD10)) %>% ungroup()
```

STOPPD11 Stop acetylcholinesterase inhibitors with a known history of persistent bradycardia (< 60 beats/min.), heart block or recurrent unexplained syncope or concurrent treatment with Drugs that reduce heart rate such as beta-blockers, digoxin, diltiazem, verapamil (risk of cardiac conduction failure, syncope and injury)
```{r}
SNPR <- mutate(SNPR,BetaDigox_DilVER = if_else(substr(SNPR$ATC,1,3)=="C07" | SNPR$ATC== "C01AA05" | SNPR$ATC== "C08DA01" |
SNPR$ATC=="C08DA51" | SNPR$ATC=="C09BB10" | SNPR$ATC =="C08DB01",1,0))
SNPR <- SNPR %>% group_by(lopnr) %>% mutate(BetaDigox_DilVERmax = max(BetaDigox_DilVER)) %>% ungroup()
```

```{r}
SNPR <- mutate(SNPR,STOPPD11 = if_else(substr(SNPR$ATC,1, 5)=="N06DA" & (SNPR$BetaDigox_DilVERmax==1 |
substr(SNPR$Diseases,1,4)=="I495" | substr(SNPR$Diseases,1,4)=="I441" | substr(SNPR$Diseases,1,4)=="I442" |
substr(SNPR$Diseases,1,4)=="I443" | substr(SNPR$Diseases,1,4)=="I455" | substr(SNPR$Diseases,1,4)=="I459" |
substr(SNPR$Diseases,1,4)=="Q246"),1,0))
SNPR <- SNPR %>% group_by(lopnr) %>% mutate(STOPPD11max = max(STOPPD11)) %>% ungroup()
```

STOPPD13 Stop Levodopa or dopamine agonists for benign essential tremor
```{r}
SNPR <- mutate(SNPR, Parkinson = if_else(substr(SNPR$Diseases,1,3)=="G20"| substr(SNPR$Diseases,1,3)=="G21" |
substr(SNPR$Diseases,1,4)=="G231" | substr(SNPR$Diseases,1,4)=="G232" | substr(SNPR$Diseases,1,4)=="G318" |
substr(SNPR$Diseases,1,4)=="G903",1,0))
SNPR <- SNPR %>% group_by(lopnr) %>% mutate(Parkinsonmax = max(Parkinson)) %>% ungroup()
```

```{r}
SNPR <- mutate(SNPR,STOPPD13 = if_else(substr(SNPR$ATC,1,4)=="N04B"& substr(SNPR$Diseases,1,4)=="G251" & Parkinsonmax == 0,1,0))
SNPR <- SNPR %>% group_by(lopnr) %>% mutate(STOPPD13max = max(STOPPD13)) %>% ungroup()
```

STOPPD14 Stop first-generation antihistamines (safer, less toxic antihistamines now widely available).
```{r}
SNPR <- mutate(SNPR,STOPPD14= if_else(substr(SNPR$ATC,1,5)=="R06AA" | substr(SNPR$ATC,1,5) =="R06AB" | substr(SNPR$ATC,1,5)
=="R06AC" | substr(SNPR$ATC,1,5) =="R06AD" | SNPR$ATC=="R06AE01" | SNPR$ATC=="R06AE03" | SNPR$ATC=="R06AE04" | SNPR$ATC=="R06AE05" |
SNPR$ATC=="R06AE06" | SNPR$ATC=="R06AE51" | SNPR$ATC=="R06AE53" | SNPR$ATC=="R06AE55" | SNPR$ATC=="R06AX01" | SNPR$ATC=="R06AX02" |
SNPR$ATC=="R06AX03" | SNPR$ATC=="R06AX04" | SNPR$ATC=="R06AX05" | SNPR$ATC=="R06AX08" | SNPR$ATC=="R06AX09" | SNPR$ATC=="R06AX15" |
SNPR$ATC=="R06AX16" | SNPR$ATC=="R06AX17" | SNPR$ATC=="R06AX23" | SNPR$ATC=="R06AX53" | SNPR$ATC=="R06AX58" | SNPR$ATC=="N05BB01" |
SNPR$ATC=="N05BB51" | SNPR$ATC=="N07CA02" | SNPR$ATC=="N07CA52",1,0))
SNPR <- SNPR %>% group_by(lopnr) %>% mutate(STOPPD14max = max(STOPPD14)) %>% ungroup()
```

Total CNS
```{r}
SNPR <- mutate (SNPR,STOPPD_CNS= STOPPD1max + STOPPD3max + STOPPD4max + STOPPD5max + STOPPD6max + STOPPD7max + STOPPD8max+
STOPPD10max + STOPPD11max + STOPPD13max + STOPPD14max)
```

Renal disorders
STOPPE2 Direct thrombin inhibitors (e.g. dabigatran) if eGFR < 30 ml/min/1.73m2
```{r}
SNPR <- mutate(SNPR,STOPPE2 = if_else(substr(SNPR$ATC,1,5)=="B01AE"& (substr(SNPR$Diseases,1,4)=="N184" |
substr(SNPR$Diseases,1,4)=="N185" | substr(SNPR$Diseases,1,4)=="N186"),1,0))

SNPR <- SNPR %>% group_by(lopnr) %>% mutate(STOPPE2max = max(STOPPE2)) %>% ungroup()
```

STOPPE3 Factor Xa inhibitors (e.g. rivaroxaban, apixaban) if eGFR < 15 ml/min/1.73m2
```{r}
SNPR <- mutate(SNPR,STOPPE3 = if_else(substr(SNPR$ATC,1,5)=="B01AF"& (substr(SNPR$Diseases,1,4)=="N185" |
substr(SNPR$Diseases,1,3)=="N186"),1,0))

SNPR <- SNPR %>% group_by(lopnr) %>% mutate(STOPPE3max = max(STOPPE3)) %>% ungroup()
```

STOPPE6 Metformin if eGFR < 30 ml/min/1.73m2
```{r}
SNPR <- mutate(SNPR,STOPPE6 = if_else((SNPR$ATC=="A10BA02" | SNPR$ATC=="A10BD02" | SNPR$ATC=="A10BD03" | SNPR$ATC=="A10BD05" |
SNPR$ATC=="A10BD07"| SNPR$ATC=="A10BD08" | SNPR$ATC=="A10BD10" | SNPR$ATC=="A10BD11" | SNPR$ATC=="A10BD13" | SNPR$ATC=="A10BD14"|
SNPR$ATC=="A10BD15" | SNPR$ATC=="A10BD16" | SNPR$ATC=="A10BD17" | SNPR$ATC=="A10BD18" | SNPR$ATC=="A10BD22") &
(substr(SNPR$Diseases,1,4)=="N184" | substr(SNPR$Diseases,1,4)=="N185" | substr(SNPR$Diseases,1,4)=="N186"),1,0))

SNPR <- SNPR %>% group_by(lopnr) %>% mutate(STOPPE6max = max(STOPPE6)) %>% ungroup()
```

Total STOPP E renal
```{r}
SNPR <- mutate (SNPR,STOPPE_Renal = STOPPE2max + STOPPE3max + STOPPE6max)
```

GI disorders

STOPPF1: Prochlorperazine or metoclopramide with Parkinsonism
```{r}

SNPR <- mutate(SNPR,STOPPF1 = if_else((SNPR$ATC=="N05AB04" | SNPR$ATC=="A03FA01") & Parkinsonmax == 1,1,0))
SNPR <- SNPR %>% group_by(lopnr) %>% mutate(STOPPF1max = max(STOPPF1)) %>% ungroup()
```

Total STOPP F GI disorders
```{r}
SNPR <- mutate (SNPR,STOPPF_GI= STOPPF1max)
```

STOPP G Respiratory system

STOPPG1 Theophylline as monotherapy for COPD
```{r}
SNPR <- mutate(SNPR,Inhalant = if_else(substr(SNPR$ATC,1,4)=="R03A"  |substr(SNPR$ATC,1,5)=="R03BA" | substr(SNPR$ATC,1,5)=="R03BB", 1,0))
SNPR <- SNPR %>% group_by(lopnr) %>% mutate(Inhalantmax = max(Inhalant)) %>% ungroup()
```

```{r}
SNPR <- mutate(SNPR,STOPPG1 = if_else(SNPR$ATC=="R03DA04" & Inhalantmax == 0 & (substr(SNPR$Diseases,1,3)=="J40" | substr(SNPR$Diseases,1,3)=="J41" | substr(SNPR$Diseases,1,3)=="J42" | substr(SNPR$Diseases,1,3)=="J43" | substr(SNPR$Diseases,1,3)=="J44"),1,0))

SNPR <- SNPR %>% group_by(lopnr) %>% mutate(STOPPG1max = max(STOPPG1)) %>% ungroup()
```

STOPPG3 Anti-muscarinic bronchodilators (e.g. ipratropium, tiotropium) with a history of narrow angle glaucoma (may exacerbate glaucoma) or bladder outflow obstruction
```{r}
SNPR <- mutate(SNPR,STOPPG3 = if_else(SNPR$ATC=="R03DA04" & (substr(SNPR$Diseases,1,4)=="H402" | substr(SNPR$Diseases,1,3)=="N40" | substr(SNPR$Diseases,1,3)=="R33"),1,0))

SNPR <- SNPR %>% group_by(lopnr) %>% mutate(STOPPG3max = max(STOPPG3)) %>% ungroup()
```

STOPPG4 Benzodiazepines with acute or chronic respiratory failure i.e. pO2 < 8.0 kPa ± pCO2 > 6.5 kPa
```{r}
SNPR <- mutate(SNPR,STOPPG4 = if_else((substr(SNPR$ATC,1,5)=="N05BA" | substr(SNPR$ATC,1,5)=="N05CD" | substr(SNPR$ATC,1,5)=="N05CF" | substr(SNPR$ATC,1,5)=="N03AE") & substr(SNPR$Diseases,1,3)=="J96",1,0))

SNPR <- SNPR %>% group_by(lopnr) %>% mutate(STOPPG4max = max(STOPPG4)) %>% ungroup()
```

Total STOPP G Respiratory
```{r}
SNPR <- mutate (SNPR,STOPPG_Resp = STOPPG1max + STOPPG3max + STOPPG4max)
```

STOPP H Musculoskeletal system

STOPPH1 Non-steroidal anti-inflammatory drug (NSAID) other than COX-2 selective agents with history of peptic ulcer disease or gastrointestinal bleeding, unless with concurrent PPI or H2 antagonist
```{r}
SNPR <- mutate(SNPR,H2antagonist = if_else(substr(SNPR$ATC,1,5)=="A02BA"  |substr(SNPR$ATC,1,5)=="A02BC", 1,0))
SNPR <- SNPR %>% group_by(lopnr) %>% mutate(H2antagonistmax = max(H2antagonist)) %>% ungroup()
```

```{r}
SNPR <- mutate(SNPR,STOPPH1 = if_else((substr(SNPR$ATC,1,5)=="M01AA" | substr(SNPR$ATC,1,5)=="M01AB" | substr(SNPR$ATC,1,5)=="M01AC" | substr(SNPR$ATC,1,5)=="M01AE" | substr(SNPR$ATC,1,5)=="M01AG" | substr(SNPR$ATC,1,5)=="M01AX" | substr(SNPR$ATC,1,5)=="M01BA" | substr(SNPR$ATC,1,5)=="N02BA") & H2antagonistmax == 0 & (substr(SNPR$Diseases,1,4)=="K221" | substr(SNPR$Diseases,1,3)=="K25" | substr(SNPR$Diseases,1,3)=="K26" | substr(SNPR$Diseases,1,3)=="K27" | substr(SNPR$Diseases,1,3)=="K28" | substr(SNPR$Diseases,1,4)=="K920" | substr(SNPR$Diseases,1,4)=="K921" | substr(SNPR$Diseases,1,4)=="K922"),1,0))
SNPR <- SNPR %>% group_by(lopnr) %>% mutate(STOPPH1max = max(STOPPH1)) %>% ungroup()
```

STOPPH4 Long-term corticosteroids (>3 months) as monotherapy for rheumatoid arthrtitis
```{r}
SNPR <- mutate(SNPR,DMARD = if_else(substr(SNPR$ATC,1,5)=="P01BA"  |substr(SNPR$ATC,1,5)=="M01CB" | substr(SNPR$ATC,1,5)=="M01CC" | SNPR$ATC=="L04AX01" | SNPR$ATC=="L04AX03" | SNPR$ATC=="L04AA13" | SNPR$ATC=="L04AD01" | SNPR$ATC=="A07EC01", 1,0))
SNPR <- SNPR %>% group_by(lopnr) %>% mutate(DMARDmax = max(DMARD)) %>% ungroup()
```

```{r}
SNPR <- mutate(SNPR,STOPPH4 = if_else((substr(SNPR$ATC,1,5)=="H02AB" | SNPR$ATC=="H02BX01") & DMARDmax == 0 & (substr(SNPR$Diseases,1,3)=="M05" | substr(SNPR$Diseases,1,3)=="M06"),1,0))
SNPR <- SNPR %>% group_by(lopnr) %>% mutate(STOPPH4max = max(STOPPH4)) %>% ungroup()
```

STOPPH5 Corticosteroids (other than periodic intra-articular injections for mono-articular pain) for osteoarthritis
```{r}
SNPR <- mutate(SNPR,STOPPH5 = if_else(((substr(SNPR$ATC,1,5)=="H02AB" & SNPR$ATC!="H02AB12") | SNPR$ATC=="H02BX01" | substr(SNPR$ATC,1,5)=="M01BA") &
(substr(SNPR$Diseases,1,3)=="M15" | substr(SNPR$Diseases,1,3)=="M16" | substr(SNPR$Diseases,1,3)=="M17" | substr(SNPR$Diseases,1,3)=="M18" |  substr(SNPR$Diseases,1,3)=="M19" | substr(SNPR$Diseases,1,3)=="M47"),1,0))
SNPR <- SNPR %>% group_by(lopnr) %>% mutate(STOPPH5max = max(STOPPH5)) %>% ungroup()
```

STOPPH7 COX-2 selective NSAIDs with concurrent cardiovascular disease
```{r}
SNPR <- mutate(SNPR,STOPPH7 = if_else(substr(SNPR$ATC,1,5)=="M01AH" & (substr(SNPR$Diseases,1,3)=="I20" | substr(SNPR$Diseases,1,3)=="I21" | substr(SNPR$Diseases,1,3)=="I22" | substr(SNPR$Diseases,1,3)=="I24" | substr(SNPR$Diseases,1,3)=="I25" | substr(SNPR$Diseases,1,4)=="Z955" | substr(SNPR$Diseases,1,4)=="Z951"),1,0))
SNPR <- SNPR %>% group_by(lopnr) %>% mutate(STOPPH7max = max(STOPPH7)) %>% ungroup()
```

STOPPH8 Stop NSAID with concurrent corticosteroids without PPI prophylaxis (increased risk of peptic ulcer disease).
```{r}
SNPR <- mutate(SNPR, Corticosteroids  = ifelse(substr(SNPR$ATC,1,4)=="H02A" | substr(SNPR$ATC,1,4)=="H02B" | SNPR$ATC=="N02CB01",1,0))
SNPR <- SNPR %>% group_by(lopnr) %>% mutate(Corticosteroidsmax = max(Corticosteroids)) %>% ungroup()
```

```{r}

SNPR <- mutate(SNPR,STOPPH8 = if_else((substr(SNPR$ATC,1,4)=="M01A" | substr(SNPR$ATC,1,5)=="N02BA" | substr(SNPR$ATC,1,5)=="M01BA" | SNPR$ATC=="N02CB01" | SNPR$ATC=="H02BX01") & Corticosteroidsmax ==1 & PPImax==0,1,0))
SNPR <- SNPR %>% group_by(lopnr) %>% mutate(STOPPH8max = max(STOPPH8)) %>% ungroup()
```

STOPPH9 Stop Oral bisphosphonates AND upper gastrointestinal disease or upper gastrointestinal bleeding.
```{r}
SNPR <- mutate(SNPR,STOPPH9 = if_else((SNPR$ATC=="M05BA01" | SNPR$ATC=="M05BA02" | SNPR$ATC=="M05BA04" | SNPR$ATC=="M05BA05" | SNPR$ATC=="M05BA06"|SNPR$ATC=="M05BA07"|substr(SNPR$ATC,1,5)=="M05BB") & (substr(SNPR$Diseases,1,2)=="K2" | substr(SNPR$Diseases,1,3)=="K30"| substr(SNPR$Diseases,1,3)=="K31" | substr(SNPR$Diseases,1,4)=="R101" | substr(SNPR$Diseases,1,3)=="R12" | substr(SNPR$Diseases,1,3)=="R13" | substr(SNPR$Diseases,1,4)=="K920" | substr(SNPR$Diseases,1,4)=="K921"| substr(SNPR$Diseases,1,4)=="K922"), 1,0))
SNPR <- SNPR %>% group_by(lopnr) %>% mutate(STOPPH9max = max(STOPPH9)) %>% ungroup()
```

Total STOPP H Musculoskeletal
```{r}
SNPR <- mutate (SNPR,STOPPH_Musculo = STOPPH1max + STOPPH4max + STOPPH5max + STOPPH7max + STOPPH8max + STOPPH9max)
```

STOPP I Urogenital system

STOPPI1 Antimuscarinic drugs with dementia, or chronic cognitive impairment (risk of increased confusion, agitation) or narrow-angle glaucoma (risk of acute exacerbation of glaucoma), or chronic prostatism (risk of urinary retention).
```{r}
SNPR <- mutate(SNPR, STOPPI1 = ifelse ((SNPR$ATC=="G04BD01" | SNPR$ATC=="G04BD02" | SNPR$ATC=="G04BD03" | SNPR$ATC=="G04BD04" | SNPR$ATC=="G04BD05" | SNPR$ATC=="G04BD06" | SNPR$ATC=="G04BD07" | SNPR$ATC=="G04BD08" | SNPR$ATC=="G04BD09" | SNPR$ATC=="G04BD10" | SNPR$ATC=="G04BD11") & (substr(SNPR$Diseases,1,3)=="F00" | substr(SNPR$Diseases,1,3)=="F01" |  substr(SNPR$Diseases,1,3)=="F02" | substr(SNPR$Diseases,1,3)=="F03" |  substr(SNPR$Diseases,1,3)=="G30" | substr(SNPR$Diseases,1,4)=="G310" | substr(SNPR$Diseases,1,4)=="G311" |  substr(SNPR$Diseases,1,4)=="G318"  |  substr(SNPR$Diseases,1,4)=="H402" | substr(SNPR$Diseases,1,3)=="N40" |  substr(SNPR$Diseases,1,3)=="R33"),1,0))
SNPR <- SNPR %>% group_by(lopnr) %>% mutate(STOPPI1max = max(STOPPI1)) %>% ungroup()
```

STOPPI2 Selective alpha-1 selective alpha blockers in those with symptomatic orthostatic hypotension or micturition syncope.
```{r}
SNPR <- mutate(SNPR, STOPPI2 = ifelse ((substr(SNPR$ATC,1,5)=="G04CA" | substr(SNPR$ATC,1,5)=="C02CA" | SNPR$ATC=="C02LE01") & substr(SNPR$Diseases,1,3)=="R55",1,0))
SNPR <- SNPR %>% group_by(lopnr) %>% mutate(STOPPI2max = max(STOPPI2)) %>% ungroup()
```

Total STOPP I Uro
```{r}
SNPR <- mutate (SNPR,STOPPI_Uro = STOPPI1max + STOPPI2max)
```

STOPP J Endrocrinology

STOPPJ1 Sulphonylureas with a long duration of action (e.g. glibenclamide, chlorpropamide, glimepiride) with type 2 diabetes mellitus
```{r}
SNPR <- mutate(SNPR, STOPPJ1 = ifelse ((SNPR$ATC=="A01BB01" | SNPR$ATC=="A10BB02" | SNPR$ATC=="A10BB12"| SNPR$ATC=="A10BD06" | SNPR$ATC=="A10BD04") & substr(SNPR$Diseases,1,3)=="E11",1,0))
SNPR <- SNPR %>% group_by(lopnr) %>% mutate(STOPPJ1max = max(STOPPJ1)) %>% ungroup()
```

STOPPJ2 Thiazolidenediones (e.g. rosiglitazone, pioglitazone) in patients with heart failure
```{r}
SNPR <- mutate(SNPR, STOPPJ2 = ifelse ((substr(SNPR$ATC,1,5)=="A10BG" | SNPR$ATC=="A10BD04" | SNPR$ATC=="A10BD03"| SNPR$ATC=="A10BD06" | SNPR$ATC=="A10BD05" | SNPR$ATC=="A10BD09" | SNPR$ATC=="A10BD12") & (substr(SNPR$Diseases,1,3)=="I50" | substr(SNPR$Diseases,1,4)=="I110" | substr(SNPR$Diseases,1,4)=="I130" | substr(SNPR$Diseases,1,4)=="I132"),1,0))
SNPR <- SNPR %>% group_by(lopnr) %>% mutate(STOPPJ2max = max(STOPPJ2)) %>% ungroup()
```

STOPPJ4 Oestrogens with a history of breast cancer or venous thromboembolism
```{r}
SNPR <- mutate(SNPR, STOPPJ4 = ifelse ((substr(SNPR$ATC,1,5)=="G03AA" | substr(SNPR$ATC,1,5)=="G03AB" | substr(SNPR$ATC,1,4)=="G03C"| substr(SNPR$ATC,1,5)=="G03EA"| substr(SNPR$ATC,1,5)=="G03EB" | substr(SNPR$ATC,1,4)=="G03F"| substr(SNPR$ATC,1,5)=="G03HB"| substr(SNPR$ATC,1,5)=="G03XC" | SNPR$ATC=="G02BB01"| substr(SNPR$ATC,1,5)=="L02AA") & (substr(SNPR$Diseases,1,3)=="C50" | substr(SNPR$Diseases,1,3)=="D05" | substr(SNPR$Diseases,1,4)=="I802" | substr(SNPR$Diseases,1,3)=="I81" |  substr(SNPR$Diseases,1,3)=="I82" | substr(SNPR$Diseases,1,3)=="I26"),1,0))
SNPR <- SNPR %>% group_by(lopnr) %>% mutate(STOPPJ4max = max(STOPPJ4)) %>% ungroup()
```

STOPPJ5 Stop oral oestrogens without progestogen in patients with intact uterus (risk of endometrial cancer)
```{r}
SNPR <- mutate(SNPR,Hysterectomy= if_else(substr(SNPR$Diseases,1,4)=="Z907",1,0))
SNPR <- SNPR %>% group_by(lopnr) %>% mutate(Hysterectomymax = max(Hysterectomy)) %>% ungroup()
```

```{r}
SNPR <- mutate(SNPR, STOPPJ5_without= if_else(substr(SNPR$ATC,1,5)=="G03AC" | substr(SNPR$ATC,1,4)=="G03D" | substr(SNPR$ATC,1,5)=="L02AB",1,0))
SNPR <- SNPR %>% group_by(lopnr) %>% mutate(STOPPJ5_withoutmax = max(STOPPJ5_without)) %>% ungroup()
```

```{r}
SNPR <- mutate(SNPR, STOPPJ5= if_else(SNPR$STOPPJ5_withoutmax == 0 & (substr(SNPR$ATC,1,4)=="G03C" | substr(SNPR$ATC,1,5)=="G03EA" | substr(SNPR$ATC,1,5)=="G03XC" | substr(SNPR$ATC,1,5)=="G03HB" | substr(SNPR$ATC,1,5)=="L02AA") & SNPR$sex=="female" & SNPR$Hysterectomymax==0,1,0))
SNPR <- SNPR %>% group_by(lopnr) %>% mutate(STOPPJ5max = max(STOPPJ5)) %>% ungroup()
```

STOPPJ6 Androgens (male sex hormones) in the absence of primary or secondary hypogonadism
```{r}
SNPR <- mutate(SNPR,Hypogonadism= if_else(substr(SNPR$Diseases,1,4)=="E291" | substr(SNPR$Diseases,1,4)=="E895" | substr(SNPR$Diseases,1,4)=="Q980" | substr(SNPR$Diseases,1,4)=="Q981" | substr(SNPR$Diseases,1,4)=="Q982" | substr(SNPR$Diseases,1,4)=="Q984",1,0))
SNPR <- SNPR %>% group_by(lopnr) %>% mutate(Hypogonadismmax = max(Hypogonadism)) %>% ungroup()
```

```{r}
SNPR <- mutate(SNPR, STOPPJ6= if_else(substr(SNPR$ATC,1,4)=="G03B" & Hypogonadismmax ==0,1,0))
SNPR <- SNPR %>% group_by(lopnr) %>% mutate(STOPPJ6max = max(STOPPJ6)) %>% ungroup()

```

Total STOPP J Endocrino
```{r}
SNPR <- mutate (SNPR,STOPPJ_Endocrino=STOPPJ1max+STOPPJ2max+STOPPJ4max+STOPPJ5max+STOPPJ6max)
```

STOPPK Drugs that increase risk of falling
STOPPK1: Stop benzodiazepines (sedative, may cause reduced sensorium, impair balance).
```{r}
SNPR <- mutate(SNPR,STOPPK1= if_else(substr(SNPR$ATC,1,5)=="N03AE" | substr(SNPR$ATC,1,5)=="N05BA" | substr(SNPR$ATC,1,5)=="N05CF" |
substr(SNPR$ATC,1,5)=="N05CD",1,0))
SNPR <- SNPR %>% group_by(lopnr) %>% mutate(STOPPK1max = max(STOPPK1)) %>% ungroup()
```

STOPPK2: Stop neuroleptic Drugs (may cause gait dyspraxia, Parkinsonism).
```{r}
SNPR <- mutate(SNPR, STOPPK2= if_else(substr(SNPR$ATC,1,4)=="N05A" & substr(SNPR$ATC,1,5)!="N05AN",1,0))
SNPR <- SNPR %>% group_by(lopnr) %>% mutate(STOPPK2max = max(STOPPK2)) %>% ungroup()
```

STOPPK3: Vasodilator drugs (e.g. alpha-1 receptor blockers, calcium channel blockers, long-acting nitrates, ACE inhibitors, angiotensin I receptor blockers,) with persistent postural hypotension i.e. recurrent drop in systolic blood pressure ≥ 20mmHg
```{r}
SNPR <- mutate(SNPR, STOPPK3= if_else((substr(SNPR$ATC,1,5)=="G04CA"| substr(SNPR$ATC,1,5)=="C02CA"| SNPR$ATC=="C02LE01"|
substr(SNPR$ATC,1,5)=="C07AG"| substr(SNPR$ATC,1,5)=="C07BG"| substr(SNPR$ATC,1,5)=="C07CG"| substr(SNPR$ATC,1,3)=="C08"|
substr(SNPR$ATC,1,3)=="C09"| substr(SNPR$ATC,1,5)=="C01DA") & substr(SNPR$Diseases,1,4)=="I951",1,0))
SNPR <- SNPR %>% group_by(lopnr) %>% mutate(STOPPK3max = max(STOPPK3)) %>% ungroup()
```

STOPPK4 Stop hypnotic Drugs e.g. zopiclone, zolpidem, zaleplon (may cause protracted daytime sedation, ataxia).
```{r}
SNPR <- mutate(SNPR, STOPPK4 = if_else(substr(SNPR$ATC,1,5)=="N05CF",1,0))
SNPR <- SNPR %>% group_by(lopnr) %>% mutate(STOPPK4max = max(STOPPK4)) %>% ungroup()
```

##Total STOPP K Drugs that increase falling
```{r}
SNPR <- mutate (SNPR,STOPPK_Fall=STOPPK1max+STOPPK2max+STOPPK3max+STOPPK4max)
```

STOPPL Analgesic drugs
STOPPL1 Use of regular (as distinct from PRN) opioids without concomitant laxative
```{r}
SNPR <- mutate(SNPR,Laxatives= if_else(substr(SNPR$ATC,1,4)=="A06A" | SNPR$ATC=="A02AA04",1,0))
SNPR <- SNPR %>% group_by(lopnr) %>% mutate(Laxativesmax = max(Laxatives)) %>% ungroup()
```

```{r}
SNPR <- mutate(SNPR, STOPPL1 = if_else((substr(SNPR$ATC,1,4)=="N02A" | SNPR$ATC=="R05DA04"| SNPR$ATC=="N07BC01"|
SNPR$ATC=="N07BC02"| SNPR$ATC=="N07BC06") & Laxativesmax==0,1,0))
SNPR <- SNPR %>% group_by(lopnr) %>% mutate(STOPPL1max = max(STOPPL1)) %>% ungroup()
```

##Total STOPP L Analgesic drugs
```{r}
SNPR <- mutate (SNPR,STOPPL_Analgesic=STOPPL1max)
```

STOPPM Anticholinergic burden

STOPP M1 Stop concomitant use of two or more Drugs with antimuscarinic/anticholinergic properties
```{r}
SNPR <- mutate(SNPR, STOPPMdrug = ifelse(SNPR$ATC=="R06AE03" | SNPR$ATC==" R06AE04" |SNPR$ATC=="R06AE05" |
substr(SNPR$ATC,1,5)=="R06AA" | substr(SNPR$ATC,1,5)=="R06AB" | substr(SNPR$ATC,1,5)=="R06AC" | substr(SNPR$ATC,1,5)=="R06AD"  |
SNPR$ATC=="R06AE01" | SNPR$ATC=="R06AE03" | SNPR$ATC=="R06AE04" | SNPR$ATC=="R06AE05" | SNPR$ATC=="R06AE06" | SNPR$ATC=="R06AE51" |
SNPR$ATC=="R06AE53" | SNPR$ATC=="R06AE55" | SNPR$ATC=="R06AX01" | SNPR$ATC=="R06AX02" | SNPR$ATC=="R06AX03" | SNPR$ATC=="R06AX04"
|SNPR$ATC=="R06AX05" | SNPR$ATC=="R06AX08" | SNPR$ATC=="R06AX09" | SNPR$ATC=="R06AX15" | SNPR$ATC=="R06AX16" | SNPR$ATC=="R06AX17" |
SNPR$ATC=="R06AX23" | SNPR$ATC=="R06AX53" | SNPR$ATC=="R06AX58" | SNPR$ATC=="N05BB01" | SNPR$ATC=="N05BB51" | SNPR$ATC=="N07CA02" |
SNPR$ATC=="N07CA52" | SNPR$ATC=="G04BD01" | SNPR$ATC=="G04BD02" | SNPR$ATC=="G04BD03" | SNPR$ATC=="G04BD04" | SNPR$ATC=="G04BD05" |
SNPR$ATC=="G04BD06" | SNPR$ATC=="G04BD07" | SNPR$ATC=="G04BD08" | SNPR$ATC=="G04BD09" | SNPR$ATC=="G04BD10" | SNPR$ATC=="G04BD11" |
substr(SNPR$ATC,1,5)=="N06AA" | substr(SNPR$ATC,1,4)=="A03B" | substr(SNPR$ATC,1,5)=="A03AA" | substr(SNPR$ATC,1,5)=="A03AB" |
SNPR$ATC=="A04AD01" | SNPR$ATC=="M03BX02" | substr(SNPR$ATC,1,4)=="N04A" | SNPR$ATC=="N05AA01" | SNPR$ATC=="N05AH02" |
SNPR$ATC=="N05AB02" | SNPR$ATC=="N05AA02" | SNPR$ATC=="N05AC02" | SNPR$ATC=="N05AA04" | SNPR$ATC=="N05AF04",1,0))
```

```{r}
#Code a variable that defines only one Index_Disease per lopnr (FirstDis). Because now the diseases are duplicated depending on
#drugs.
SNPR <- SNPR  %>% group_by(lopnr) %>%
  mutate(IndexDiseases_first = first(IndexDiseases))  %>% ungroup()
SNPR <- SNPR %>%
  mutate(FirstDis = case_when(
    IndexDiseases != IndexDiseases_first ~ 0,
    IndexDiseases == IndexDiseases_first ~ 1))
SNPR$FirstDis <- factor(SNPR$FirstDis,label = c("Duplicate", "Original"))
```

```{r}
#We create a subset of the original dataframe where only the rows with the original or one Index of disease are included.
SNPRChol <- SNPR[SNPR$FirstDis == "Original", ]
```

```{r}
#We run in this subset the code below: Calculating the sum of anticholinergic drugs
SNPRChol <- SNPRChol %>% group_by(lopnr) %>% mutate(STOPPMdrugsum = sum(STOPPMdrug)) %>% ungroup()
SNPRChol <- SNPRChol %>% group_by(lopnr) %>% mutate(STOPPM1 = STOPPMdrugsum > 1)  %>% ungroup()
```

```{r}
#We add this newly created variable STOPP1 back to the original dataframe. The sum will be copied
SNPR$newvariable  <- SNPRChol[match(SNPR$lopnr,SNPRChol$lopnr),"STOPPM1"]
SNPR$STOPPM1 <- SNPR$newvariable$STOPPM1
```
```

````
## STOPP TOTAL Anticholinergic burden
```{r}
SNPR <- mutate(SNPR, STOPPM_Cholineoverload = STOPPM1)
```

```{r}
rm(SNPRChol)
```

################################################################################
# START criteria

## STARTA cardiovascular system.
### STARTA1 Start vitamin K antagonists or direct thrombin inhibitors or factor Xa inhibitors in the presence of chronic atrial fibrillation.
```{r}
SNPR <- mutate(SNPR, AFtreatment = if_else(substr(SNPR$ATC,1,5)=="B01AA"  | substr(SNPR$ATC,1,5) == "B01AB" | substr(SNPR$ATC,1,5) =="B01AE" | substr(SNPR$ATC,1,5) == "B01AF",1,0))
SNPR <- SNPR %>% group_by(lopnr) %>% mutate(AFtreatmentmax = max(AFtreatment)) %>% ungroup()
```

```{r}
SNPR <- mutate(SNPR, STARTA1= if_else(substr(SNPR$Diseases,1,3)=="I48" & SNPR$AFtreatmentmax==0, 1,0))
SNPR <- SNPR %>% group_by(lopnr) %>% mutate(STARTA1max = max(STARTA1)) %>% ungroup()
```

### STARTA2 Start aspirin (75 mg - 160 mg once daily) in the presence of chronic atrial fibrillation, where Vitamin K antagonists or direct thrombin inhibitors or factor Xa inhibitors are contraindicated.

```{r}
SNPR <- mutate(SNPR, Acetylcar= if_else(SNPR$ATC=="B01AC06" | SNPR$ATC=="B01AC56" | SNPR$ATC=="B01AC08",1,0))
SNPR <- SNPR %>% group_by(lopnr) %>% mutate(Acetylcarmax = max(Acetylcar)) %>% ungroup()
```

```{r}
SNPR <- mutate(SNPR, STARTA2= if_else(substr(SNPR$Diseases,1,3)=="I48" & SNPR$AFtreatmentmax==0 & SNPR$Acetylcarmax==0,1,0))
SNPR <- SNPR %>% group_by(lopnr) %>% mutate(STARTA2max = max(STARTA2)) %>% ungroup()
```

### STARTA3 Start antiplatelet therapy (aspirin or clopidogrel or prasugrel or ticagrelor) with a documented history of coronary, cerebral or peripheral vascular disease.

```{r}
SNPR <- mutate(SNPR,AF = if_else(substr(SNPR$Diseases,1,3)=="I48",1,0))
SNPR <- SNPR %>% group_by(lopnr) %>% mutate(AFmax = max(AF)) %>% ungroup()
```

```{r}
SNPR <- mutate(SNPR, Acetylcarbasalateclopidrogel= if_else(SNPR$ATC=="B01AC06" | SNPR$ATC=="B01AC08" | SNPR$ATC=="B01AC22" | SNPR$ATC=="B01AC24" | SNPR$ATC=="B01AC04",1,0))
SNPR <- SNPR %>% group_by(lopnr) %>% mutate(Acetylcarbasalateclopidrogelmax = max(Acetylcarbasalateclopidrogel)) %>% ungroup()
```

```{r}
SNPR <- mutate(SNPR, STARTA3= if_else(SNPR$Acetylcarbasalateclopidrogelmax == 0 & (substr(SNPR$Diseases,1,3)=="I20" | substr(SNPR$Diseases,1,3)=="I21" | substr(SNPR$Diseases,1,3)=="I22" | substr(SNPR$Diseases,1,3)=="I24" | substr(SNPR$Diseases,1,3)=="I25" | substr(SNPR$Diseases,1,4)=="Z955" | substr(SNPR$Diseases,1,4)=="Z951" | substr(SNPR$Diseases,1,4)=="Z958" | substr(SNPR$Diseases,1,3)=="I63" | substr(SNPR$Diseases,1,3)=="I64" | substr(SNPR$Diseases,1,3)=="I65" | substr(SNPR$Diseases,1,3)=="I66" | substr(SNPR$Diseases,1,4)=="I739" | substr(SNPR$Diseases,1,3)=="I74" | substr(SNPR$Diseases,1,3)=="G45") & AFmax==0,1,0))
SNPR <- SNPR %>% group_by(lopnr) %>% mutate(STARTA3max = max(STARTA3)) %>% ungroup()
```

### STARTA5 Start statin therapy with a documented history of coronary, cerebral or peripheral vascular disease, unless the patient's status is end-of-life or age is > 85 years.

```{r}
SNPR <- mutate(SNPR, Statin= if_else(substr(SNPR$ATC,1,5)=="C10AA" | substr(SNPR$ATC,1,4)=="C10B",1,0))
SNPR <- SNPR %>% group_by(lopnr) %>% mutate(Statinmax = max(Statin)) %>% ungroup()
```

```{r}
SNPR <- mutate(SNPR, STARTA5= if_else(SNPR$Statinmax == 0 & (substr(SNPR$Diseases,1,3)=="I20" |  substr(SNPR$Diseases,1,3)=="I21" | substr(SNPR$Diseases,1,3)=="I22" |  substr(SNPR$Diseases,1,3)=="I24" |  substr(SNPR$Diseases,1,3)=="I25" | substr(SNPR$Diseases,1,4)=="Z955" | substr(SNPR$Diseases,1,4)=="Z951" | substr(SNPR$Diseases,1,4)=="Z958" | substr(SNPR$Diseases,1,3)=="I63" |  substr(SNPR$Diseases,1,3)=="I64" |  substr(SNPR$Diseases,1,3)=="I65" | substr(SNPR$Diseases,1,3)=="I66" |  substr(SNPR$Diseases,1,4)=="I739" |  substr(SNPR$Diseases,1,3)=="I74" | substr(SNPR$Diseases,1,3)=="G45") & SNPR$age < 85,1,0))
SNPR <- SNPR %>% group_by(lopnr) %>% mutate(STARTA5max = max(STARTA5)) %>% ungroup()

```

### STARTA6 Start ACE inhibitor with systolic heart failure and/or documented coronary artery disease.
```{r}
SNPR <- mutate(SNPR,ACEi_ANGII= if_else(substr(SNPR$ATC,1,3)=="C09",1,0))
SNPR <- SNPR %>% group_by(lopnr) %>% mutate(ACEi_ANGIImax = max(ACEi_ANGII)) %>% ungroup()
```

```{r}
SNPR <- mutate(SNPR,STARTA6= if_else(SNPR$ACEi_ANGIImax == 0 & (substr (SNPR$Diseases,1,4)=="I502" | substr(SNPR$Diseases,1,3)=="I20" | substr(SNPR$Diseases,1,3)=="I21" |   substr(SNPR$Diseases,1,3)=="I22" | substr(SNPR$Diseases,1,3)=="I24" |  substr(SNPR$Diseases,1,3)=="I25" |  substr(SNPR$Diseases,1,4)=="Z955" |substr(SNPR$Diseases,1,4)=="Z951"),1,0))
SNPR <- SNPR %>% group_by(lopnr) %>% mutate(STARTA6max = max(STARTA6)) %>% ungroup()
```

### STARTA7: Start beta-blocker with ischaemic heart disease.
```{r}
SNPR <- mutate(SNPR,Betablocker=if_else(substr(SNPR$ATC,1,3)=="C07",1,0))
SNPR <- SNPR %>% group_by(lopnr) %>% mutate(Betablockermax = max(Betablocker)) %>% ungroup()
```

```{r}
````
````

````
SNPR <- mutate(SNPR,STARTA7= if_else(SNPR$Betablockermax == 0 & (substr(SNPR$Diseases,1,3)=="I20" |
substr(SNPR$Diseases,1,3)=="I21" |   substr(SNPR$Diseases,1,3)=="I22" | substr(SNPR$Diseases,1,3)=="I24" |
substr(SNPR$Diseases,1,3)=="I25" |  substr(SNPR$Diseases,1,4)=="Z955" |  substr(SNPR$Diseases,1,4)=="Z951"),1,0))
SNPR <- SNPR %>% group_by(lopnr) %>% mutate(STARTA7max = max(STARTA7)) %>% ungroup()
```

## Total START A cardiovascular
```{r}
SNPR <- mutate (SNPR,STARTA_Cardiovascular=STARTA1max+STARTA2max+STARTA3max+STARTA5max+STARTA6max+STARTA7max)
```

##STARTB Respiratory system
### STARTB1 Regular inhaled B2 agonist or antimuscarinic bronchodilator (e.g. ipratropium, tiotropium) for mild to moderate asthma
or COPD
```{r}
SNPR <- mutate(SNPR,Broncho= if_else(substr(SNPR$ATC,1,5)=="R03AC" | substr(SNPR$ATC,1,5)=="R03BB",1,0))
SNPR <- SNPR %>% group_by(lopnr) %>% mutate(Bronchomax = max(Broncho)) %>% ungroup()
```

```{r}
SNPR <- mutate(SNPR,STARTB1= if_else(SNPR$Bronchomax == 0 & (substr(SNPR$Diseases,1,3)=="J40" |  substr(SNPR$Diseases,1,3)=="J41" |
substr(SNPR$Diseases,1,3)=="J42" | substr(SNPR$Diseases,1,3)=="J43" |  substr(SNPR$Diseases,1,3)=="J44" |
substr(SNPR$Diseases,1,4)=="J452" | substr(SNPR$Diseases,1,4)=="J453" | substr(SNPR$Diseases,1,4)=="J454"),1,0))
SNPR <- SNPR %>% group_by(lopnr) %>% mutate(STARTB1max = max(STARTB1)) %>% ungroup()

```

### STARTB3 Home continuous oxygen with documented chronic hypoxaemia (i.e. pO2 < 8.0 kPa or 60 mmHg or SaO2 < 89%)
```{r}
SNPR <- mutate(SNPR,Oxygen= if_else(SNPR$ATC=="V03AN01",1,0))
SNPR <- SNPR %>% group_by(lopnr) %>% mutate(Oxygenmax = max(Oxygen)) %>% ungroup()
```

```{r}
SNPR <- mutate(SNPR,STARTB3= if_else(SNPR$Oxygenmax == 0 & (substr(SNPR$Diseases,1,5)=="J9691" |  substr(SNPR$Diseases,1,5)=="J9611"
|   substr(SNPR$Diseases,1,5)=="R0902"),1,0))
SNPR <- SNPR %>% group_by(lopnr) %>% mutate(STARTB3max = max(STARTB3)) %>% ungroup()
```

##STARTB Total Respiratory system
```{r}
SNPR <- mutate (SNPR,STARTB_Resp=STARTB1max+STARTB3max)
```

## START C CNS

###STARTC2 Non-TCA antidepressant drug in the presence of persistent major depressive symptoms.
```{r}
SNPR <- mutate(SNPR,nonTCA= if_else(substr(SNPR$ATC,1,5)=="N06AB" | substr(SNPR$ATC,1,5)=="N06AF"|substr(SNPR$ATC,1,5)=="N06AG" |
substr(SNPR$ATC,1,5)=="N06AX" | SNPR$ATC=="N06CA03",1,0))
SNPR <- SNPR %>% group_by(lopnr) %>% mutate(nonTCAmax = max(nonTCA)) %>% ungroup()
```

```{r}
SNPR <- mutate(SNPR,STARTC2= if_else(SNPR$nonTCAmax == 0 & substr(SNPR$Diseases,1,3)=="F33",1,0))
SNPR <- SNPR %>% group_by(lopnr) %>% mutate(STARTC2max = max(STARTC2)) %>% ungroup()
```

###STARTC3 Acetylcholinesterase inhibitor (e.g. donepezil, rivastigmine, galantamine) for mild-moderate Alzheimer’s dementia or Lewy
Body dementia (rivastigmine).
```{r}
SNPR <- mutate(SNPR,rivastigmine= if_else(SNPR$ATC=="N06DA03",1,0))
SNPR <- SNPR %>% group_by(lopnr) %>% mutate(rivastigminemax = max(rivastigmine)) %>% ungroup()

SNPR <- mutate(SNPR,STARTC3= if_else(SNPR$rivastigminemax == 0 & substr(SNPR$Diseases,1,4)=="G318",1,0))
SNPR <- SNPR %>% group_by(lopnr) %>% mutate(STARTC3max = max(STARTC3)) %>% ungroup()
```

###STARTC4 Topical prostaglandin, prostamide or beta-blocker for primary open-angle glaucoma.
```{r}
SNPR <- mutate(SNPR,Prostagl= if_else(substr(SNPR$ATC,1,5)=="S01EE" | substr(SNPR$ATC,1,5)=="S01ED",1,0))
SNPR <- SNPR %>% group_by(lopnr) %>% mutate(Prostaglmax = max(Prostagl)) %>% ungroup()
```

```{r}
SNPR <- mutate(SNPR,STARTC4= if_else(SNPR$Prostaglmax == 0 & substr(SNPR$Diseases,1,5)=="H4011",1,0))
SNPR <- SNPR %>% group_by(lopnr) %>% mutate(STARTC4max = max(STARTC4)) %>% ungroup()
```

###STARTC5 Selective serotonin reuptake inhibitor (or SNRI or pregabalin if SSRI contraindicated) for persistent severe anxiety that
interferes with independent functioning.
```{r}
SNPR <- mutate(SNPR,SSRI= if_else(substr(SNPR$ATC,1,5)=="N06AB" | SNPR$ATC=="N06AX16" | SNPR$ATC=="N06AX21" | SNPR$ATC=="N03AX16"|
SNPR$ATC=="N06CA03",1,0))
SNPR <- SNPR %>% group_by(lopnr) %>% mutate(SSRImax = max(SSRI)) %>% ungroup()
```

```{r}
SNPR <- mutate(SNPR,STARTC5= if_else(SNPR$SSRImax == 0 & substr(SNPR$Diseases,1,4)=="F419",1,0))
SNPR <- SNPR %>% group_by(lopnr) %>% mutate(STARTC5max = max(STARTC5)) %>% ungroup()
```

###STARTC6 Dopamine agonist (ropinirole or pramipexole or rotigotine) for Restless Legs Syndrome, once iron deficiency and severe
renal failure have been excluded.
```{r}
SNPR <- mutate(SNPR,Dopamin= if_else(SNPR$ATC=="N04BC04" | SNPR$ATC=="N04BC05" | SNPR$ATC=="N04BC09",1,0))
SNPR <- SNPR %>% group_by(lopnr) %>% mutate(Dopaminmax = max(Dopamin)) %>% ungroup()

SNPR <- mutate(SNPR,IronRenal= if_else(substr(SNPR$Diseases,1,4)=="N184" | substr(SNPR$Diseases,1,4)=="N185" |
substr(SNPR$Diseases,1,4)=="N186" | substr(SNPR$Diseases,1,3)=="E61" | substr(SNPR$Diseases,1,3)=="D50",1,0))
SNPR <- SNPR %>% group_by(lopnr) %>% mutate(IronRenalmax = max(IronRenal)) %>% ungroup()

SNPR <- mutate(SNPR,STARTC6= if_else(SNPR$Dopaminmax == 0 & SNPR$IronRenalmax == 0 & substr(SNPR$Diseases,1,5)=="H4011",1,0))
SNPR <- SNPR %>% group_by(lopnr) %>% mutate(STARTC6max = max(STARTC6)) %>% ungroup()
````

````
```

##Total STARTC CNS
```{r}
SNPR <- mutate (SNPR,STARTC_CNS=STARTC2max+STARTC3max+STARTC4max+STARTC5max+STARTC6max)
```

## START D GI Disorders

### STARTD2 Start fibre supplements (e.g. bran, ispaghula, methylcellulose, sterculia) for diverticulosis with a history of constipation.
```{r}
SNPR <- mutate(SNPR,Fiber = if_else(substr(SNPR$ATC,1,5)=="A06AC",1,0))
SNPR <- SNPR %>% group_by(lopnr) %>% mutate(Fibermax = max(Fiber)) %>% ungroup()

SNPR <- mutate(SNPR,Constipation = if_else(substr(SNPR$Diseases,1,4)=="K590",1,0))
SNPR <- SNPR %>% group_by(lopnr) %>% mutate(Constipationmax = max(Constipation)) %>% ungroup()
```

```{r}
SNPR <- mutate(SNPR,STARTD2 = if_else(substr(SNPR$Diseases,1,3)=="K57" & SNPR$Constipationmax==1 & SNPR$Fibermax==0,1,0))
SNPR <- SNPR %>% group_by(lopnr) %>% mutate(STARTD2max = max(STARTD2)) %>% ungroup()
```

## Total START D GI Disorders
```{r}
SNPR <- mutate (SNPR,STARTD_GI=STARTD2max)
```

## START E Locomotor system
### STARTE2 Start bisphosphonates and vitamin D and calcium in patients taking long-term systemic corticosteroid therapy.
```{r}
SNPR <- mutate(SNPR, Bifosfonates= if_else(substr(SNPR$ATC,1,5)=="M05BA" | substr(SNPR$ATC,1,5)=="M05BB" |
substr(SNPR$ATC,1,5)=="A11CC" | substr(SNPR$ATC,1,5)=="A11CB" | SNPR$ATC=="A11GB01" | SNPR$ATC=="A11AA02" |
substr(SNPR$ATC,1,5)=="A12AA" | substr(SNPR$ATC,1,5)=="A12AX",1,0))
SNPR <- SNPR %>% group_by(lopnr) %>% mutate(Bifosfonatesmax = max(Bifosfonates)) %>% ungroup()
```

```{r}
SNPR <- mutate(SNPR,STARTE2= if_else(substr(SNPR$ATC,1,5)=="H02AB" & SNPR$Bifosfonatesmax == 0,1,0))
SNPR <- SNPR %>% group_by(lopnr) %>% mutate(STARTE2max = max(STARTE2)) %>% ungroup()
```

### STARTE3 Start vitamin D and calcium supplement in patients with known osteoporosis and/or previous fragility fracture(s) and/or (Bone Mineral Density T-scores more than -2.5 in multiple sites).
```{r}
SNPR <- mutate(SNPR, Calcium_VitD=  if_else(substr(SNPR$ATC,1,5)=="A11CC" | substr(SNPR$ATC,1,5)=="A11CB" | SNPR$ATC=="A11GB01" |
SNPR$ATC=="A11AA02" | substr(SNPR$ATC,1,5)=="A12AA" | substr(SNPR$ATC,1,5)=="A12AX" |SNPR$ATC=="M05BB01" | SNPR$ATC=="M05BB02" |
SNPR$ATC=="M05BB03" | SNPR$ATC=="M05BB04" | SNPR$ATC=="M05BB05" | SNPR$ATC=="M05BB06" | SNPR$ATC=="M05BB07" |
SNPR$ATC=="M05BB08",1,0))
SNPR <- SNPR %>% group_by(lopnr) %>% mutate(Calcium_VitDmax = max(Calcium_VitD)) %>% ungroup()
```

```{r}
SNPR <- mutate(SNPR,STARTE3= if_else(SNPR$Calcium_VitDmax == 0 & (substr(SNPR$Diseases,1,3)=="M80" |
substr(SNPR$Diseases,1,3)=="M81" | substr(SNPR$Diseases,1,3)=="S12" | substr(SNPR$Diseases,1,3)=="S22" |
substr(SNPR$Diseases,1,3)=="S32" | substr(SNPR$Diseases,1,3)=="S42" | substr(SNPR$Diseases,1,3)=="S52" |
substr(SNPR$Diseases,1,3)=="S62" | substr(SNPR$Diseases,1,3)=="S72" | substr(SNPR$Diseases,1,3)=="S82" |
substr(SNPR$Diseases,1,3)=="S92" | substr(SNPR$Diseases,1,3)=="T02" | substr(SNPR$Diseases,1,3)=="T08" |
substr(SNPR$Diseases,1,3)=="T10" | substr(SNPR$Diseases,1,3)=="T12" | substr(SNPR$Diseases,1,4)=="T142"),1,0))
SNPR <- SNPR %>% group_by(lopnr) %>% mutate(STARTE3max = max(STARTE3)) %>% ungroup()
```

### STARTE4 Bone anti-resorptive or anabolic therapy (e.g. bisphosphonate, strontium ranelate, teriparatide, denosumab) in patients with documented osteoporosis, where no pharmacological or clinical status contraindication exists (Bone Mineral Density T-scores -> 2.5 in multiple sites) and/or previous history of fragility fracture(s).
```{r}
SNPR <- mutate(SNPR, Anabolic= if_else(substr(SNPR$ATC,1,4)=="M05B" | SNPR$ATC=="H05AA02",1,0))
SNPR <- SNPR %>% group_by(lopnr) %>% mutate(Anabolicmax = max(Anabolic)) %>% ungroup()
```

```{r}
SNPR <- mutate(SNPR, Fractures= if_else(substr(SNPR$Diseases,1,3)=="S12" | substr(SNPR$Diseases,1,3)=="S22" |
substr(SNPR$Diseases,1,3)=="S32" | substr(SNPR$Diseases,1,3)=="S42" | substr(SNPR$Diseases,1,3)=="S52" |
substr(SNPR$Diseases,1,3)=="S62" | substr(SNPR$Diseases,1,3)=="S72" | substr(SNPR$Diseases,1,3)=="S82" |
substr(SNPR$Diseases,1,3)=="S92" | substr(SNPR$Diseases,1,3)=="T02" | substr(SNPR$Diseases,1,3)=="T08" |
substr(SNPR$Diseases,1,3)=="T10" | substr(SNPR$Diseases,1,3)=="T12" | substr(SNPR$Diseases,1,4)=="T142",1,0))
SNPR <- SNPR %>% group_by(lopnr) %>% mutate(Fracturesmax = max(Fractures)) %>% ungroup()
```

```{r}
SNPR <- mutate(SNPR,STARTE4= if_else(SNPR$Anabolicmax == 0 & SNPR$Fracturesmax == 1 & (substr(SNPR$Diseases,1,3)=="M80" |
substr(SNPR$Diseases,1,3)=="M81"),1,0))
SNPR <- SNPR %>% group_by(lopnr) %>% mutate(STARTE4max = max(STARTE4)) %>% ungroup()
```

### STARTE5 Vitamin D supplement in older people who are housebound or experiencing falls or with osteopenia (Bone Mineral Density T-score is > -1.0 but < -2.5 in multiple sites).
```{r}
SNPR <- mutate(SNPR, VitD=  if_else(substr(SNPR$ATC,1,5)=="A11CC" | substr(SNPR$ATC,1,5)=="A11CB" | substr(SNPR$ATC,1,5)=="A12AX" |
SNPR$ATC=="M05BB03" | SNPR$ATC=="M05BB04" | SNPR$ATC=="M05BB05" | SNPR$ATC=="M05BB06" | SNPR$ATC=="M05BB07" |
SNPR$ATC=="M05BB08",1,0))
SNPR <- SNPR %>% group_by(lopnr) %>% mutate(VitDmax = max(VitD)) %>% ungroup()
```

```{r}
SNPR <- mutate(SNPR,STARTE5= if_else(SNPR$VitDmax == 0 & (substr(SNPR$Diseases,1,4)=="R296" | substr(SNPR$Diseases,1,4)=="R263" |
substr(SNPR$Diseases,1,3)=="W01" | substr(SNPR$Diseases,1,3)=="W05" | substr(SNPR$Diseases,1,3)=="W06" |
substr(SNPR$Diseases,1,3)=="W07" | substr(SNPR$Diseases,1,3)=="W08" | substr(SNPR$Diseases,1,3)=="W10" |
substr(SNPR$Diseases,1,3)=="W18" | substr(SNPR$Diseases,1,3)=="W19" | substr(SNPR$Diseases,1,5) == "M8580"),1,0))
SNPR <- SNPR %>% group_by(lopnr) %>% mutate(STARTE5max = max(STARTE5)) %>% ungroup()
```

### STARTE6 Start xanthine-oxidase inhibitors (e.g. allopurinol, febuxostat) with a history of recurrent episodes of gout.
````
````

````
```{r}
SNPR <- mutate(SNPR,Xanthine = if_else(substr(SNPR$ATC,1,5)=="M04AA",1,0))
SNPR <- SNPR %>% group_by(lopnr) %>% mutate(Xanthinemax = max(Xanthine)) %>% ungroup()

SNPR <- mutate(SNPR,STARTE6 = if_else(substr(SNPR$Diseases,1,3)=="M10" & SNPR$Xanthinemax == 0,1,0))
SNPR <- SNPR %>% group_by(lopnr) %>% mutate(STARTE6max = max(STARTE6)) %>% ungroup()
```

### STARTE7 Folic acid supplement in patients taking methotrexate.
```{r}
SNPR <- mutate(SNPR,Folic = if_else(substr(SNPR$ATC,1,5)=="B03BB" | substr(SNPR$ATC,1,5)=="B03AD" | SNPR$ATC=="B03AE02" |
SNPR$ATC=="B03AE01",1,0))
SNPR <- SNPR %>% group_by(lopnr) %>% mutate(Folicmax = max(Folic)) %>% ungroup()

SNPR <- mutate(SNPR,STARTE7 = if_else(SNPR$ATC=="L04AX03" & SNPR$Folicmax == 0,1,0))
SNPR <- SNPR %>% group_by(lopnr) %>% mutate(STARTE7max = max(STARTE7)) %>% ungroup()
```

## Total START E locomotor system
```{r}
SNPR <- mutate (SNPR,STARTE_Muskulo=STARTE2max+STARTE3max+STARTE4max+STARTE5max+STARTE6max+STARTE7max)
```

## STARTF Endocrino
### STARTF1 ACE inhibitor or Angiotensin Receptor Blocker (if intolerant of ACE inhibitor) in diabetes with evidence of renal
disease i.e. dipstick proteinuria or microalbuminuria (>30mg/24 hours) with or without serum biochemical renal impairment.
```{r}
SNPR <- mutate(SNPR,ACE = if_else(substr(SNPR$ATC,1,4)=="C09A" | substr(SNPR$ATC,1,4)=="C09B" | substr(SNPR$ATC,1,4)=="C09C" |
substr(SNPR$ATC,1,4)=="C09D",1,0))
SNPR <- SNPR %>% group_by(lopnr) %>% mutate(ACEmax = max(ACE)) %>% ungroup()
```

```{r}
SNPR <- mutate(SNPR,Diabetes = if_else(substr(SNPR$Diseases,1,3)=="E10" | substr(SNPR$Diseases,1,3)=="E11" |
substr(SNPR$Diseases,1,3)=="E12" | substr(SNPR$ATC,1,3)=="E13" | substr(SNPR$Diseases,1,3)=="E14",1,0))
SNPR <- SNPR %>% group_by(lopnr) %>% mutate(Diabetesmax = max(Diabetes)) %>% ungroup()
```

```{r}
SNPR <- mutate(SNPR,STARTF1= if_else(SNPR$ACEmax == 0 & ((SNPR$Diabetesmax == 1 & substr(SNPR$Diseases,1,3)=="R80") |
(substr(SNPR$Diseases,1,4)=="E102" | substr(SNPR$Diseases,1,4)=="E112" | substr(SNPR$Diseases,1,4)=="E122" |
substr(SNPR$Diseases,1,4)=="E132" | substr(SNPR$Diseases,1,4)=="E142" | substr(SNPR$Diseases,1,4)=="N083")),1,0))
SNPR <- SNPR %>% group_by(lopnr) %>% mutate(STARTF1max = max(STARTF1)) %>% ungroup()
```

## Total START F Endocrino
```{r}
SNPR <- mutate (SNPR,STARTF_Endo=STARTF1max)
```

##STARTH Analgesics
###STARTH2 Laxatives in patients receiving opioids regularly. <- This is actually the same as STOPPL1
```{r}
SNPR <- mutate(SNPR,STARTH2= if_else(SNPR$Laxativesmax == 0 & (substr(SNPR$ATC,1,4)=="N02A" | SNPR$ATC=="R05DA04"|
SNPR$ATC=="N07BC01"| SNPR$ATC=="N07BC02"| SNPR$ATC=="N07BC06") ,1,0))
SNPR <- SNPR %>% group_by(lopnr) %>% mutate(STARTH2max = max(STARTH2)) %>% ungroup()
```

## Total START H Analgesics
```{r}
SNPR <- mutate (SNPR,STARTH_Analg=STARTH2max)
```

##Make bleeding risk variables specifically for adverse outcome association analysis
```{r}
SNPR <- mutate(SNPR,Bleedingrisk = if_else( Anticoagulantiamax == 1 | substr(SNPR$ATC,1,5) == "B01AB" | substr(SNPR$ATC,1,5) ==
"B01AC" | substr(SNPR$ATC,1,4)=="M01A" | substr(SNPR$ATC,1,5)=="N02BA" | substr(SNPR$ATC,1,5)=="N06AB" | ATC=="N06DX02" |
substr(SNPR$ATC,1,5)=="H02AB",1 ,0))
SNPR <- SNPR %>% group_by(lopnr ) %>% mutate (Bleedingriskmax = max(Bleedingrisk)) %>% ungroup()
```

# This is the code to have one row per ID/lopnr.
```{r}
#remove object from workspace because you don't need it anymore
SNPR <- SNPR[, -which(names(SNPR) %in% c ("Anticoagulantia", "Anticoagulantiamax","Antiplatelet","Antiplateletmax", "Inhalant",
"Inhalantmax", "Laxatives", "Laxativesmax", "AF",
"ACEi_ANGII","ACEi_ANGIImax","Acetylcarbasalateclopidrogel","Acetylcarbasalateclopidrogelmax", "Betablocker", "Betablockermax",
"Broncho", "ACE", "ACEmax","Diabetes","Diabetesmax","Oxygen", "IndexDiseases_first","FirstDis", "Diseases", "IndexDiseases",
"newvariable","AFtreatment","Acetylcarmax","AFmax","Statin", "Statinmax"))]

SNPR<-SNPR[order(SNPR$lopnr), ]
SNPR$Duplicate <- duplicated(SNPR$lopnr)
SNPRnew <- SNPR[SNPR$Duplicate == FALSE,]
rm(SNPR)
SNPRnew <- filter(SNPRnew, Atrial_fibrillation==1 & age >= 65 & numberdiseases >= 2)
```

## Totals and binary versions
```{r}
SNPRnew <- mutate(SNPRnew,STARTtotal=STARTA_Cardiovascular+STARTB_Resp+STARTC_CNS+STARTD_GI+STARTE_Muskulo+STARTF_Endo)
SNPRnew <-
mutate(SNPRnew,STOPPtotal=STOPPB_Cardiovascular+STOPPC_AntiplateletAnticoagulent+STOPPD_CNS+STOPPE_Renal+STOPPF_GI+STOPPG_Resp+STOPPH_
SNPRnew$STOPP=ifelse(SNPRnew$STOPPtotal >= 1, 1,0)
SNPRnew$START=ifelse(SNPRnew$STARTtotal >= 1,1,0)
SNPRnew$PIPtotal = SNPRnew$STOPPtotal + SNPRnew$STARTtotal
SNPRnew$PIPbinary = ifelse(SNPRnew$PIPtotal >= 1,1,0)
SNPRnew$PIP = ifelse(SNPRnew$STOPP == 1 & SNPRnew$START == 0, 1,
                     ifelse(SNPRnew$STOPP == 0 & SNPRnew$START == 1,2,
                            ifelse (SNPRnew$STOPP == 1 & SNPRnew$START ==1,3, 0)))

SNPRnew$PIP<- factor(SNPRnew$PIP, levels = c(0,1,2,3), labels = c("No PIP","STOPP","START","PIP"))

SNPRnew$STOPPB_Cardiovascular_binary= ifelse(SNPRnew$STOPPB_Cardiovascular  >= 1,1,0)
SNPRnew$STOPPC_AntiplatAnticoag_binary= ifelse(SNPRnew$STOPPC_AntiplateletAnticoagulent  >= 1,1,0)
````
````

```
SNPRnew$STOPPD_CNS_binary=ifelse(SNPRnew$STOPPD_CNS  >= 1,1,0)
SNPRnew$STOPPE_Renal_binary= ifelse(SNPRnew$STOPPE_Renal >=1,1,0)
SNPRnew$STOPPF_GI_binary=ifelse(SNPRnew$STOPPF_GI  >= 1,1,0)
SNPRnew$STOPPG_Resp_binary=ifelse(SNPRnew$STOPPG_Resp  >= 1,1,0)
SNPRnew$STOPPH_Musculo_binary=ifelse(SNPRnew$STOPPH_Musculo  >= 1,1,0)
SNPRnew$STOPPSTOPPI_Uro_binary=ifelse(SNPRnew$STOPPI_Uro  >= 1,1,0)
SNPRnew$STOPPJ_Endocrino_binary=ifelse(SNPRnew$STOPPJ_Endocrino  >= 1,1,0)
SNPRnew$STOPPK_Fall_binary= ifelse(SNPRnew$STOPPK_Fall  >= 1,1,0)
SNPRnew$STOPPL_Analgesic_binary=ifelse(SNPRnew$STOPPL_Analgesic  >= 1,1,0)
SNPRnew$STOPPM_Cholineoverload_binary=ifelse(SNPRnew$STOPPM_Cholineoverload  >= 1,1,0)

SNPRnew$STARTA_Cardiovascular_binary = ifelse(SNPRnew$STARTA_Cardiovascular >= 1,1,0)
SNPRnew$STARTB_Resp_binary = ifelse(SNPRnew$STARTB_Resp >= 1,1,0)
SNPRnew$STARTC_CNS_binary = ifelse(SNPRnew$STARTC_CNS >= 1,1,0)
SNPRnew$STARTD_GI_binary = ifelse(SNPRnew$STARTD_GI >= 1,1,0)
SNPRnew$STARTE_Muskulo_binary = ifelse(SNPRnew$STARTE_Muskulo >= 1,1,0)
SNPRnew$STARTF_Endo_binary = ifelse(SNPRnew$STARTF_Endo >= 1,1,0)
SNPRnew$STARTH_Analg_binary = ifelse(SNPRnew$STARTH_Analg >= 1,1,0)
SNPRnew$Bleedingrisk_binary = SNPRnew$Bleedingriskmax
```
