# Supplementary material for: Potentially inappropriate prescribing in polymedicated older adults with atrial fibrillation and multimorbidity: a Swedish national register-based cohort study
Source: Front Pharmacol. 2024 Sep 10;15:1476464. doi: 10.3389/fphar.2024.1476464 (PMC11420530; doi:10.3389/fphar.2024.1476464)
Supplement: Supplementary file 2 [file DataSheet4.docx]

**Potentially inappropriate prescribing in polymedicated older adults with atrial fibrillation and multimorbidity: A Swedish national register-based cohort study**

Cheima Amrouch^1,2^, Davide Liborio Vetrano^3,4^, Cecilia Damiano^5^, Lu Dai^3^, Amaia Calderón-Larrañaga^3,4^, Maxim Grymonprez^2,6^, Marco Proietti^7,8^, Gregory Y.H. Lip^9,10^, Søren P. Johnsen^10^, Jonas W. Wastesson^3,11^, Kristina Johnell^11^, Delphine De Smedt^1^*, Mirko Petrovic^2^*, *on behalf of the AFFIRMO project
** Shared last-author

*1* Department of Public Health and Primary Care, Ghent University, Ghent, Belgium
*2* Department of Internal Medicine and Paediatrics, Ghent University, Ghent, Belgium
*3* Aging Research Center, Department of Neurobiology, Care Sciences and Society, Karolinska Institutet and Stockholm University, Stockholm, Sweden
*4* Stockholm Gerontology Research Center, Stockholm, Sweden
*5* Department of Cardiovascular, Endocrine-Metaboslic Diseases and Aging, Istituto Superiore di Sanità, Rome, Italy
*6* Department of Bioanalysis, Pharmaceutical Care Unit, Ghent University, Ghent, Belgium
*7* Department of Clinical Sciences and Community Health, University of Milan, Milan, Italy
*8* Division of Subacute Care, IRCCS Istituti Clinici Scientifici Maugeri, Milan, Italy
*9* Liverpool Centre for Cardiovascular Science at University of Liverpool, Liverpool John Moores University and Liverpool Heart & Chest Hospital, Liverpool, UK
*10* Danish Center for Health Services Research, Department of Clinical Medicine, Aalborg University, Aalborg, Denmark
*11* Department of Medical Epidemiology and Biostatistics, Karolinska Institutet, Stockholm, Sweden

Table S1. Prevalence of reduced STOPP/START version 2 criteria and categories stratified by polypharmacy level.

|  | **Overall** | **No polypharmacy** | **Polypharmacy** | **Excessive polypharmacy** |
| --- | --- | --- | --- | --- |
|  | 203042 | 54943 (27.1) | 95212 (46.9) | 52887 (26.0) |
| **PIP (%)** |  |  |  |  |
| Appropriate prescribing (no PIM or PPO) (%) | 54428 (26.8) | 18047 (32.8) | 28919 (30.4) | 7462 (14.1) |
| STOPP (only PIM) (%) | 29513 (14.5) | 2130 (3.9) | 14597 (15.3) | 12786 (24.2) |
| START (only PPO) (%) | 68906 (33.9) | 29603 (53.9) | 29642 (31.1) | 9661 (18.3) |
| PIM&PPO (%) | 50195 (24.7) | 5163 (9.4) | 22054 (23.2) | 22978 (43.4) |
| **STOPPB Cardiovascular system (%)** | **6942 (3.4)** | 802 (1.5) | 3358 (3.5) | 2782 (5.3) |
| STOPPB1: Digoxin for heart failure with normal systolic ventricular function (%) | 0 (0) | 0 (0) | 0 (0) | 0 (0) |
| STOPPB3: β-blocker in combination with verapamil or diltiazem (%) | 1399 (0.7) | 112 (0.2) | 663 (0.7) | 624 (1.2) |
| STOPPB4: β-blocker with bradycardia (< 50/min), type II heart block or complete heart block (%) | 4662 (2.3) | 649 (1.2) | 2321 (2.4) | 1692 (3.2) |
| STOPPB7: Loop diuretic for dependent ankle oedema without clinical, biochemical evidence or radiological evidence of heart failure, liver failure, nephrotic syndrome, or renal failure (%) | 0 (0) | 0 (0) | 0 (0) | 0 (0) |
| STOPPB8: Thiazide diuretic with current significant hypokalaemia (i.e., serum K+ < 3.0 mmol/l), hyponatraemia (i.e., serum Na+ < 130 mmol/l) hypercalcaemia (i.e., corrected serum calcium > 2.65 mmol/l) or with a history of gout (%) | 352 (0.2) | 26 (0.0) | 159 (0.2) | 167 (0.3) |
| STOPPB9: Loop diuretic for treatment of hypertension with concurrent urinary incontinence (%) | 557 (0.3) | 15 (0.0) | 226 (0.2) | 316 (0.6) |
| STOPPB11: ACE inhibitors or Angiotensin Receptor Blockers in patients with hyperkalaemia (%) | 0 (0) | 0 (0) | 0 (0) | 0 (0) |
| STOPPB13: Phosphodiesterase type-5 inhibitors (e.g., sildenafil, tadalafil, vardenafil) in severe heart failure characterised by hypotension i.e., systolic BP < 90 mmHg, or concurrent nitrate therapy for angina (%) | 50 (0.0) | 0 (0.0) | 23 (0.0) | 27 (0.1) |
| **STOPPC Antiplatelet/Anticoagulants (%)** | **10151 (5.0)** | 873 (1.6) | 4671 (4.9) | 4607 (8.7) |
| STOPPC2: Aspirin with a history of peptic ulcer disease without concomitant PPI (%) | 195 (0.1) | 39 (0.1) | 96 (0.1) | 60 (0.1) |
| STOPPC3: Aspirin, clopidogrel, dipyridamole, vitamin K antagonists, direct thrombin inhibitors or factor Xa inhibitors with concurrent significant bleeding risk, i.e., uncontrolled severe hypertension, bleeding diathesis, recent non-trivial spontaneous bleeding) (%) | 3330 (1.6) | 496 (0.9) | 1641 (1.7) | 1193 (2.3) |
| STOPPC4: Aspirin plus clopidogrel as secondary stroke prevention, unless the patient has a coronary stent(s) inserted in the previous 12 months or concurrent acute coronary syndrome or has a high grade symptomatic carotid arterial stenosis (%) | 169 (0.1) | 2 (0.0) | 64 (0.1) | 103 (0.2) |
| STOPPC5: Aspirin in combination with vitamin K antagonist, direct thrombin inhibitor or factor Xa inhibitors in patients with chronic atrial fibrillation (%) | 3974 (2.0) | 168 (0.3) | 1690 (1.8) | 2116 (4.0) |
| STOPPC7: Ticlopidine in any circumstances (%) | 0 (0) | 0 (0) | 0 (0) | 0 (0) |
| STOPPC10: NSAID and vitamin K antagonist, direct thrombin inhibitor or factor Xa inhibitors in combination (%) | 2491 (1.2) | 147 (0.3) | 1140 (1.2) | 1204 (2.3) |
| STOPPC11: NSAID with concurrent antiplatelet agent(s) without PPI prophylaxis (%) | 396 (0.2) | 28 (0.1) | 183 (0.2) | 185 (0.3) |
| **STOPPD Central nervous system (%)** | **51427 (25.3)** | 3940 (7.2) | 22089 (23.2) | 25398 (48.0) |
| STOPPD1: Tricyclic Antidepressants (TCAs) with dementia, narrow angle glaucoma, cardiac conduction abnormalities, prostatism, or prior history of urinary retention (%) | 507 (0.2) | 11 (0.0) | 174 (0.2) | 322 (0.6) |
| STOPPD3: Neuroleptics with moderate-marked antimuscarinic/anticholinergic effects (chlorpromazine, clozapine, flupenthixol, fluphenzine, pipothiazine, promazine, zuclopenthixol) with a history of prostatism or previous urinary retention (%) | 39 (0.0) | 1 (0.0) | 17 (0.0) | 21 (0.0) |
| STOPPD4: Selective serotonin re-uptake inhibitors (SSRI’s) with current or recent significant hyponatraemia i.e., serum Na+ < 130 mmol/l (%) | 0 (0) | 0 (0) | 0 (0) | 0 (0) |
| STOPPD5: Benzodiazepines for ≥ 4 weeks (%) | 46556 (22.9) | 3539 (6.4) | 19690 (20.7) | 23327 (44.1) |
| STOPPD6: Antipsychotics (i.e., other than quetiapine or clozapine) in those with parkinsonism or Lewy Body Disease (%) | 424 (0.2) | 15 (0.0) | 173 (0.2) | 236 (0.4) |
| STOPPD7: Anticholinergics/antimuscarinics to treat extra-pyramidal side-effects of neuroleptic medications (%) | 0 (0) | 0 (0) | 0 (0) | 0 (0) |
| STOPPD8: Anticholinergics/antimuscarinics in patients with delirium or dementia (%) | 695 (0.3) | 18 (0.0) | 233 (0.2) | 444 (0.8) |
| STOPPD10: Neuroleptics as hypnotics, unless sleep disorder is due to psychosis or dementia (%) | 0 (0) | 0 (0) | 0 (0) | 0 (0) |
| STOPPD11: Acetylcholinesterase inhibitors with a known history of persistent bradycardia (< 60 beats/min.), heart block or recurrent unexplained syncope or concurrent treatment with medications that reduce heart rate such as β-blockers, digoxin, diltiazem, verapamil (%) | 2662 (1.3) | 160 (0.3) | 1315 (1.4) | 1187 (2.2) |
| STOPPD13: Levodopa or dopamine agonists for benign essential tremor (%) | 0 (0) | 0 (0) | 0 (0) | 0 (0) |
| STOPPD14: First-generation antihistamines (%) | 4544 (2.2) | 262 (0.5) | 1619 (1.7) | 2663 (5.0) |
| **STOPPE Renal system (%)** | **101 (0.0)** | 3 (0.0) | 31 (0.0) | 67 (0.1) |
| STOPPE2: Direct thrombin inhibitors (e.g., dabigatran) if eGFR < 30 ml/min/1.73m2 (%) | 8 (0.0) | 2 (0.0) | 3 (0.0) | 3 (0.0) |
| STOPPE3: Factor Xa inhibitors (e.g., rivaroxaban, apixaban) if eGFR < 15 ml/min/1.73m2 (%) | 71 (0.0) | 1 (0.0) | 24 (0.0) | 46 (0.1) |
| STOPPE6: Metformin if eGFR < 30 ml/min/1.73m2 (%) | 22 (0.0) | 0 (0.0) | 4 (0.0) | 18 (0.0) |
| **STOPPF GI (%)** | **23 (0)** | 0 (0.0) | 4 (0.0) | 19 (0.0) |
| STOPPF1: Prochlorperazine or metoclopramide with Parkinsonism (%) | 23 (0) | 0 (0.0) | 4 (0.0) | 19 (0.0) |
| **STOPPG Respiratory system (%)** | **1206 (0.6)** | 39 (0.1) | 251 (0.3) | 916 (1.7) |
| STOPPG1: Theophylline as monotherapy for COPD (%) | 10 (0.0) | 3 (0.0) | 1 (0.0) | 6 (0.0) |
| STOPPG3: Anti-muscarinic bronchodilators (e.g., ipratropium, tiotropium) with a history of narrow angle glaucoma (may exacerbate glaucoma) or bladder outflow obstruction (%) | 14 (0.0) | 2 (0.0) | 4 (0.0) | 8 (0.0) |
| STOPPG4: Benzodiazepines with acute or chronic respiratory failure i.e., pO2 < 8.0 kPa ± pCO2 > 6.5 kPa (%) | 1186 (0.6) | 36 (0.1) | 246 (0.3) | 904 (1.7) |
| **STOPPH Musculoskeletal system (%)** | **5308 (2.6)** | 236 (0.4) | 1772 (1.9) | 3300 (6.2) |
| STOPPH1: Non-steroidal anti-inflammatory drug (NSAID) other than COX-2 selective agents with history of peptic ulcer disease or gastrointestinal bleeding, unless with concurrent PPI or H2 antagonist (%) | 38 (0.0) | 6 (0.0) | 15 (0.0) | 17 (0.0) |
| STOPPH4: Long-term corticosteroids (>3 months) as monotherapy for rheumatoid arthritis (%) | 1295 (0.6) | 65 (0.1) | 459 (0.5) | 771 (1.5) |
| STOPPH5: Corticosteroids (other than periodic intra-articular injections for mono-articular pain) for osteoarthritis (%) | 3286 (1.6) | 137 (0.2) | 1060 (1.1) | 2089 (3.9) |
| STOPPH7: COX-2 selective NSAIDs with concurrent cardiovascular disease (%) | 149 (0.1) | 4 (0.0) | 49 (0.1) | 96 (0.2) |
| STOPPH8: NSAID with concurrent corticosteroids without PPI prophylaxis (%) | 270 (0.1) | 10 (0.0) | 101 (0.1) | 159 (0.3) |
| STOPPH9: Oral bisphosphonates in patients with a current or recent history of upper gastrointestinal disease i.e., dysphagia, oesophagitis, gastritis, duodenitis, or peptic ulcer disease, or upper gastrointestinal bleeding (%) | 815 (0.4) | 31 (0.1) | 243 (0.3) | 541 (1.0) |
| **STOPPI Urogenital system (%)** | **1496 (0.7)** | 123 (0.2) | 697 (0.7) | 676 (1.3) |
| STOPPI1: Antimuscarinic medications with dementia, or chronic cognitive impairment (risk of increased confusion, agitation) or narrow-angle glaucoma (risk of acute exacerbation of glaucoma), or chronic prostatism (risk of urinary retention) (%) | 676 (0.3) | 329 (0.2) | 218 (0.4) | 129 (0.8) |
| STOPPI2: Selective alpha-1 selective alpha blockers in those with symptomatic orthostatic hypotension or micturition syncope (%) | 844 (0.4) | 422 (0.3) | 284 (0.5) | 138 (0.9) |
| **STOPPJ Endocrine system (%)** | **1947 (1.0)** | 131 (0.2) | 822 (0.9) | 994 (1.9) |
| STOPPJ1: Sulphonylureas with a long duration of action (e.g., glibenclamide, chlorpropamide, glimepiride) with type 2 diabetes mellitus (%) | 1163 (0.6) | 56 (0.1) | 512 (0.5) | 595 (1.1) |
| STOPPJ2: Thiazolidinediones (e.g., rosiglitazone, pioglitazone) in patients with heart failure (%) | 31 (0) | 1 (0.0) | 11 (0.0) | 19 (0.0) |
| STOPPJ4: Oestrogens with a history of breast cancer or venous thromboembolism (%) | 301 (0.1) | 24 (0.0) | 108 (0.1) | 169 (0.3) |
| STOPPJ5: Oral oestrogens without progestogen in patients with intact uterus (%) | 0 (0.0) | 0 (0.0) | 0 (0.0) | 0 (0.0) |
| STOPPJ6: Androgens (male sex hormones) in the absence of primary or secondary hypogonadism (%) | 459 (0.2) | 50 (0.1) | 191 (0.2) | 218 (0.4) |
| **STOPPK Fall Risk Inducing Drugs (%)** | **50687 (25.0)** | 4000 (7.3) | 21803 (22.9) | 24884 (47.1) |
| STOPPK1: Benzodiazepines (%) | 46556 (22.9) | 3539 (6.4) | 19690 (20.7) | 23327 (44.1) |
| STOPPK2: Neuroleptic medications (%) | 4182 (2.1) | 198 (0.4) | 1540 (1.6) | 2444 (4.6) |
| STOPPK3: Vasodilator drugs (e.g. alpha-1 receptor blockers, calcium channel blockers, long-acting nitrates, ACE inhibitors, angiotensin I receptor blockers, ) with persistent postural hypotension i.e. recurrent drop in systolic blood pressure ≥ 20mmHg (%) | 3459 (1.7) | 326 (0.6) | 1541 (1.6) | 1592 (3.0) |
| STOPPK4: Hypnotic Z-medications e.g., zopiclone, zolpidem, zaleplon (%) | 36207 (17.8) | 2819 (5.1) | 15351 (16.1) | 18037 (34.1) |
| **STOPPL Analgesics (%)** | **17572 (8.7)** | **1150 (2.1)** | **7107 (7.5)** | **9315 (17.6)** |
| STOPPL1: Use of regular (as distinct from PRN) opioids without concomitant laxative (%) | 17572 (8.7) | 1150 (2.1) | 7107 (7.5) | 9315 (17.6) |
| **STOPPM Choline overload (%)** | **507 (0.2)** | 5 (0.0) | 98 (0.1) | 404 (0.8) |
| STOPPM1: Concomitant use of two or more medications with antimuscarinic/anticholinergic properties (e.g., bladder antispasmodics, intestinal antispasmodics, tricyclic antidepressants, first generation antihistamines) | 507 (0.2) | 5 (0.0) | 98 (0.1) | 404 (0.8) |
| **STARTA Cardiovascular system (%)** | **96406 (47.5)** | 31435 (57.2) | 40989 (43.1) | 23982 (45.3) |
| STARTA1: Vitamin K antagonists or direct thrombin inhibitors or factor Xa inhibitors in the presence of chronic atrial fibrillation. (%) | 69611 (34.3) | 47260 (35.0) | 16772 (32.4) | 5579 (34.4) |
| STARTA2: Aspirin (75 mg – 160 mg once daily) in the presence of chronic atrial fibrillation, where Vitamin K antagonists or direct thrombin inhibitors or factor Xa inhibitors are contraindicated. (%) | 52548 (25.9) | 38611 (28.6) | 10846 (20.9) | 3091 (19.1) |
| STARTA3: Antiplatelet therapy (aspirin or clopidogrel or prasugrel or ticagrelor) with a documented history of coronary, cerebral, or peripheral vascular disease (%) | 0 (0) | 0 (0) | 0 (0) | 0 (0) |
| STARTA5: Statin therapy with a documented history of coronary, cerebral, or peripheral vascular disease, unless the patient’s status is end-of-life or age is > 85 years. (%) | 22635 (11.1) | 7637 (13.9) | 9355 (9.8) | 5643 (10.7) |
| STARTA6: Angiotensin Converting Enzyme (ACE) inhibitor with systolic heart failure and/or documented coronary artery disease (%) | 24901 (12.3) | 7058 (12.8) | 10493 (11.0) | 7350 (13.9) |
| STARTA7: β-blocker with ischaemic heart disease (%) | 16867 (8.3) | 5929 (10.8) | 7010 (7.4) | 3928 (7.4) |
| **STARTB Respiratory system (%)** | **9413 (4.6)** | 1835 (3.3) | 4014 (4.2) | 3564 (6.7) |
| STARTB1: Regular inhaled B2 agonist or antimuscarinic bronchodilator (e.g., ipratropium, tiotropium) for mild to moderate asthma or COPD (%) | 9389 (4.6) | 1834 (3.3) | 4008 (4.2) | 3547 (6.7) |
| STARTB3: Home continuous oxygen with documented chronic hypoxaemia (i.e., pO2 < 8.0 kPa or 60 mmHg or SaO2 < 89%) (%) | 36 (0.0) | 6 (0.0) | 9 (0.0) | 21 (0.0) |
| **STARTC Central nervous system (%)** | **475 (0.2)** | 129 (0.2) | 200 (0.2) | 146 (0.3) |
| STARTC2: Non-TCA antidepressant drug in the presence of persistent major depressive symptoms (%) | 475 (0.2) | 312 (0.2) | 121 (0.2) | 42 (0.3) |
| STARTC3: Acetylcholinesterase inhibitor (e.g. donepezil, rivastigmine, galantamine) for mild-moderate Alzheimer’s dementia or Lewy Body dementia (rivastigmine) | 0 (0) | 0 (0) | 0 (0) | 0 (0) |
| STARTC4: Topical prostaglandin, prostamide or β-blocker for primary open-angle glaucoma. (%) | 0 (0) | 0 (0) | 0 (0) | 0 (0) |
| STARTC5: STARTC5 Selective serotonin reuptake inhibitor (or SNRI or pregabalin if SSRI contraindicated) for persistent severe anxiety that interferes with independent functioning. | 0 (0) | 0 (0) | 0 (0) | 0 (0) |
| STARTC6: Dopamine agonist (ropinirole or pramipexole or rotigotine) for Restless Legs Syndrome, once iron deficiency and severe renal failure have been excluded. (%) | 0 (0) | 0 (0) | 0 (0) | 0 (0) |
| **STARTD Gastrointestinal system (%)** | **972 (0.5)** | 127 (0.2) | 335 (0.4) | 510 (1.0) |
| STARTD2: Fibre supplements (e.g., bran, ispaghula, methylcellulose, sterculia) for diverticulosis with a history of constipation. (%) | 972 (0.5) | 127 (0.2) | 335 (0.4) | 510 (1.0) |
| **STARTE Musculoskeletal system (%)** | **40745 (20.1)** | 8022 (14.6) | 18307 (19.2) | 14416 (27.3) |
| STARTE2: Bisphosphonates and vitamin D and calcium in patients taking long-term systemic corticosteroid therapy. (%) | 9649 (4.8) | 745 (1.4) | 3839 (4.0) | 5065 (9.6) |
| STARTE3: Vitamin D and calcium supplement in patients with known osteoporosis and/or previous fragility fracture(s) and/or (Bone Mineral Density T-scores more than -2.5 in multiple sites). (%) | 27277 (13.4) | 6427 (11.7) | 12565 (13.2) | 8285 (15.7) |
| STARTE4: Bone anti-resorptive or anabolic therapy (e.g., bisphosphonate, strontium ranelate, teriparatide, denosumab) in patients with documented osteoporosis, where no pharmacological or clinical status contraindication exists (Bone Mineral Density T-scores -> 2.5 in multiple sites) and/or previous history of fragility fracture(s). (%) | 3207 (1.6) | 418 (0.8) | 1299 (1.4) | 1490 (2.8) |
| STARTE5: Vitamin D supplement in older people who are housebound or experiencing falls or with osteopenia (Bone Mineral Density T-score is > -1.0 but < -2.5 in multiple sites). (%) | 0 (0) | 0 (0) | 0 (0) | 0 (0) |
| STARTE6: Xanthine-oxidase inhibitors (e.g., allopurinol, febuxostat) with a history of recurrent episodes of gout. (%) | 4280 (2.1) | 915 (1.7) | 1949 (2.0) | 1416 (2.7) |
| STARTE7: Folic acid supplement in patients taking methotrexate. (%) | 1036 (0.5) | 118 (0.2) | 516 (0.5) | 402 (0.8) |
| **STARTF Endocrine system (%)** | **701 (0.3)** | 75 (0.1) | 201 (0.2) | 425 (0.8) |
| STARTF1 ACE inhibitor or Angiotensin Receptor Blocker (if intolerant of ACE inhibitor) in diabetes with evidence of renal disease i.e., dipstick proteinuria or microalbuminuria (>30mg/24 hours) with or without serum biochemical renal impairment. (%) | 701 (0.3) | 75 (0.1) | 201 (0.2) | 425 (0.8) |

Table S2. Association (OR and 95%CI) between demographic factors and the START criterion A1: “PPO of OAC in older adults with atrial fibrillation”. Estimates are derived from adjusted binary logistic regression models.

|  | **OAC PPO**  **OR [95%CI]** |
| --- | --- |
| **Age** |  |
| 75-84 years | 1.03 [1.01-1.06] |
| ≥ 85 years | 1.39 [1.35-1.43] |
| **Sex** |  |
| Male sex | 0.95 [0.93-0.97] |
| **Education** |  |
| High school | *1.02 [0.99-1.04]* |
| University or more | *1.01 [0.98-1.04]* |
| **Civil status** |  |
| Unpartnered | 1.15 [1.12-1.17] |
| **Income** |  |
| Q2 | *0.99 [0.96-1.02]* |
| Q3 | *0.97 [0.94-1.00]* |
| Q4 | 0.90 [0.87-0.94] |
| Q5 | 0.88 [0.85-0.91] |
| **Polypharmacy status** |  |
| Polypharmacy | 0.34 [0.33-0.35] |
| Excessive polypharmacy | 0.27 [0.26-0.27] |

*OR= odds ratio, Q = quintile.
The reference groups are: the complement of PPO STARTA1 criterion, age range [65-74] years, female sex, elementary education, having a partner, lowest income quintile (Q1), and no polypharmacy.*

Table S3. Association (HR and 95%CI) between START criterion A1: “PPO of OAC in older adults with atrial fibrillation” and adverse health outcomes. Estimates are derived from adjusted Cox proportional regression analyses conducted over a two-year follow-up period.

|  | **OAC PPO**  **HR [95%CI]** |
| --- | --- |
| **All-cause mortality** | 2.17 [2.10-2.24] |
| **CV mortality** | 1.99 [1.89-2.08] |
| **All-cause hospitalisation** | 1.40 [1.37-1.42] |
| **CV hospitalisation** | 1.28 [1.24-1.32] |
| **Stroke** | 1.96 [1.81-2.13] |
| **Bleeding** | 1.07 [0.98-1.17] |
| **Falls** | 1.41 [1.34-1.47] |

*HR = hazard ratio, CV= cardiovascular.*Models are adjusted by sex, age, civil status, education, income and polypharmacy.
