# Supplementary material for: Potentially inappropriate prescribing in polymedicated older adults with atrial fibrillation and multimorbidity: a Swedish national register-based cohort study
Source: Front Pharmacol. 2024 Sep 10;15:1476464. doi: 10.3389/fphar.2024.1476464 (PMC11420530; doi:10.3389/fphar.2024.1476464)
Supplement: Supplementary file 3 [file DataSheet3.docx]

**Potentially inappropriate prescribing in polymedicated older adults with atrial fibrillation and multimorbidity: A Swedish national register-based cohort study**

Cheima Amrouch^1,2^, Davide Liborio Vetrano^3,4^, Cecilia Damiano^5^, Lu Dai^3^, Amaia Calderón-Larrañaga^3,4^, Maxim Grymonprez^2,6^, Marco Proietti^7,8^, Gregory Y.H. Lip^9,10^, Søren P. Johnsen^10^, Jonas W. Wastesson^3,11^, Kristina Johnell^11^, Delphine De Smedt^1^*, Mirko Petrovic^2^*, *on behalf of the AFFIRMO project
** Shared last-author

*1* Department of Public Health and Primary Care, Ghent University, Ghent, Belgium
*2* Department of Internal Medicine and Paediatrics, Ghent University, Ghent, Belgium
*3* Aging Research Center, Department of Neurobiology, Care Sciences and Society, Karolinska Institutet and Stockholm University, Stockholm, Sweden
*4* Stockholm Gerontology Research Center, Stockholm, Sweden
*5* Department of Cardiovascular, Endocrine-Metaboslic Diseases and Aging, Istituto Superiore di Sanità, Rome, Italy
*6* Department of Bioanalysis, Pharmaceutical Care Unit, Ghent University, Ghent, Belgium
*7* Department of Clinical Sciences and Community Health, University of Milan, Milan, Italy
*8* Division of Subacute Care, IRCCS Istituti Clinici Scientifici Maugeri, Milan, Italy
*9* Liverpool Centre for Cardiovascular Science at University of Liverpool, Liverpool John Moores University and Liverpool Heart & Chest Hospital, Liverpool, UK
*10* Danish Center for Health Services Research, Department of Clinical Medicine, Aalborg University, Aalborg, Denmark
*11* Department of Medical Epidemiology and Biostatistics, Karolinska Institutet, Stockholm, Sweden

The following assumptions and adjustments were necessary for the automation of the reduced STOPP/START version 2 criteria:

- First, when the criterion specified biochemical parameters, the corresponding ICD10 code was used due to lack of these variables in the registries.
- Second, if the ICD10 code did not differentiate between chronic and acute conditions, this distinction was omitted.
- Third, no account could be given to drug adherence or consumption, since only prescribed pharmacy-dispensed medications could be considered. Consequently, an assumption was made that the patient took pharmacy-dispensed medications.
- Fourth, for criteria requiring the simultaneous occurrence of medications and/or diseases, it was assumed to be applicable when both diagnosis and medications were identifiable. This assumption was required because the diagnosis date was not extracted in the dataset used in this study.

Within the STOPP category “Analgesics” only the criterion STOPPL2 *“Use of regular opioids without concomitant laxatives”* could be coded. Similarly, within the START category “Analgesics” only the criterion STARTH2 *“Laxatives in patients receiving opioids regularly”* could be coded. Upon technical translation, these two criteria are equivalent. Therefore, in our analyses, the START Analgesics (STARTH2) category was excluded, and only the STOPP Analgesics (STOPPL2) category was incorporated. This approach ensures that when identifying the proportion of patients with the co-occurrence of STOPP (PIM) and START (PPO), false positives are avoided.

None of the START categories related to *Indication of medications*, *Urogenital system* and *Vaccines* were applicable. The data source lacked necessary variables (e.g., symptoms or side-effects), specific conditions were not covered by ICD10 codes (e.g., erosive oesophagitis), and essential information (e.g. drug doses), was inaccessible. These factors limited the application of the criteria.

Table 1. An overview of the applicability of the reduced STOPP/STARTv2 criteria. The causes of inapplicability and the adjustments performed are also detailed within the table.

| **Reduced STOPP/STARTv2 criteria** | **Criteria** | **Applicability** | **Adjustments performed** |
| --- | --- | --- | --- |
| **Indication of medication** |  | | |
| **STOPPA1** | Any drug prescribed without an evidence-based clinical indication. | N | Too broad criterion |
| **STOPPA2** | Any drug prescribed beyond the recommended duration, where treatment duration is well defined. | N | Too broad criterion |
| **STOPPA3** | Any duplicate drug class prescription e.g. two concurrent NSAIDs, SSRIs, loop diuretics, ACE inhibitors, anticoagulants (optimisation of monotherapy within a single drug class should be observed prior to considering a new agent). | N | Too broad criterion |
| **Cardiovascular system** |  | | |
| **STOPPB1** | Digoxin for heart failure with normal systolic ventricular function | Y | ICD10-code I50.3 was used for heart failure with systolic ventricular function |
| **STOPPB2** | Verapamil or diltiazem with NYHA Class III or IV heart failure | N | No ICD-10 code for NYHA Class III-IV |
| **STOPPB3** | Beta-blocker in combination with verapamil or diltiazem | Y |  |
| **STOPPB4** | Beta blocker with bradycardia (< 50/min), type II heart block or complete heart block | Y | Had to use ICD-10 code instead of heart rate measurement |
| **STOPPB5** | Amiodarone as first-line antiarrhythmic therapy in supraventricular tachyarrhythmias | N | Cannot code for first-line treatment |
| **STOPPB6** | Loop diuretic as first-line treatment for hypertension | N | Cannot code for first-line treatment |
| **STOPPB7** | Loop diuretic for dependent ankle oedema without clinical, biochemical evidence or radiological evidence of heart failure, liver failure, nephrotic syndrome or renal failure | Y | Included only the ICD10 codes |
| **STOPPB8** | Thiazide diuretic with current significant hypokalaemia (i.e. serum K+ < 3.0 mmol/l), hyponatraemia (i.e. serum Na+ < 130 mmol/l) hypercalcaemia (i.e. corrected serum calcium > 2.65 mmol/l) or with a history of gout | Y | Have to use ICD10 code instead of laboratory values |
| **STOPPB9** | Loop diuretic for treatment of hypertension with concurrent urinary incontinence | Y |  |
| **STOPPB10** | Centrally-acting antihypertensives (e.g. methyldopa, clonidine, moxonidine, rilmenidine, guanfacine), unless clear intolerance of, or lack of efficacy with, other classes of antihypertensives | N | Cannot code for intolerance |
| **STOPPB11** | ACE inhibitors or Angiotensin Receptor Blockers in patients with hyperkalaemia. | Y | Have to use ICD10 code instead of laboratory values |
| **STOPPB12** | Aldosterone antagonists (e.g. spironolactone, eplerenone) with concurrent potassium-conserving drugs (e.g. ACEI’s, ARB’s, amiloride, triamterene) without monitoring of serum potassium | N | Cannot code for monitoring of serum potassium |
| **STOPPB13** | Phosphodiesterase type-5 inhibitors (e.g. sildenafil, tadalafil, vardenafil) in severe heart failure characterised by hypotension i.e. systolic BP < 90 mmHg, or concurrent nitrate therapy for angina | Y | We could code half of the criterium "Use of Phosphodiesterase type V inhibitors with nitrate therapy for angina |
| **Antiplatelet / anticoagulant drugs** |  | | |
| **STOPPC1** | Long-term aspirin at doses greater than 160mg per day | N | Drug doses were not accessible |
| **STOPPC2** | Aspirin with a past history of peptic ulcer disease without concomitant PPI | Y |  |
| **STOPPC3** | Aspirin, clopidogrel, dipyridamole, vitamin K antagonists, direct thrombin inhibitors or factor Xa inhibitors with concurrent significant bleeding risk, i.e. uncontrolled severe hypertension, bleeding diathesis, recent non-trivial spontaneous bleeding) | Y |  |
| **STOPPC4** | Aspirin plus clopidogrel as secondary stroke prevention, unless the patient has a coronary stent(s) inserted in the previous 12 months or concurrent acute coronary syndrome or has a high grade symptomatic carotid arterial stenosis | Y | Cannot code for "high grade symptomatic" or “coronary stent inserted in the previous 12 months” |
| **STOPPC5** | Aspirin in combination with vitamin K antagonist, direct thrombin inhibitor or factor Xa inhibitors in patients with chronic atrial fibrillation | Y |  |
| **STOPPC6** | Antiplatelet agents with vitamin K antagonist, direct thrombin inhibitor or factor Xa inhibitors in patients with stable coronary, cerebrovascular or peripheral arterial disease | N | Cannot code for stable status of the conditions |
| **STOPPC7** | Ticlopidine in any circumstances | Y |  |
| **STOPPC8** | Vitamin K antagonist, direct thrombin inhibitor or factor Xa inhibitors for first deep venous thrombosis without continuing provoking risk factors (e.g. thrombophilia) for > 6 months | N | Provoking risk factors not defined |
| **STOPPC9** | Vitamin K antagonist, direct thrombin inhibitor or factor Xa inhibitors for first pulmonary embolus without continuing provoking risk factors (e.g. thrombophilia) for > 12 months | N | Provoking risk factors not defined |
| **STOPPC10** | NSAID and vitamin K antagonist, direct thrombin inhibitor or factor Xa inhibitors in combination | Y |  |
| **STOPPC11** | NSAID with concurrent antiplatelet agent(s) without PPI prophylaxis | Y |  |
| **Central nervous system and psychotropic drugs** |  | | |
| **STOPPD1** | TriCyclic Antidepressants (TCAs) with dementia, narrow angle glaucoma, cardiac conduction abnormalities, prostatism, or prior history of urinary retention | Y |  |
| **STOPPD2** | Initiation of TriCyclic Antidepressants (TCAs) as first-line antidepressant treatment | N | Cannot code for first-line treatment |
| **STOPPD3** | Neuroleptics with moderate-marked antimuscarinic/anticholinergic effects (chlorpromazine, clozapine, flupenthixol, fluphenzine, pipothiazine, promazine, zuclopenthixol) with a history of prostatism or previous urinary retention | Y |  |
| **STOPPD4** | Selective serotonin re-uptake inhibitors (SSRI’s) with current or recent significant hyponatraemia i.e. serum Na+ < 130 mmol/l | Y | Use ICD10 code instead of sodium levels |
| **STOPPD5** | Benzodiazepines for ≥ 4 weeks | Y | Drugs dispensed for 90 days prior to baseline was collected and the use of benzodiazepines for a month is not unusual, hence the specific period of ≥ 4 weeks was not explicitly integrated in the code |
| **STOPPD6** | Antipsychotics (i.e. other than quetiapine or clozapine) in those with parkinsonism or Lewy Body Disease | Y |  |
| **STOPPD7** | Anticholinergics/antimuscarinics to treat extra-pyramidal side-effects of neuroleptic medications | Y |  |
| **STOPPD8** | Anticholinergics/antimuscarinics in patients with delirium or dementia | Y |  |
| **STOPPD9** | Neuroleptic antipsychotic in patients with behavioural and psychological symptoms of dementia (BPSD) unless symptoms are severe and other non-pharmacological treatments have failed | N | Cannot code for "behavioural and psychological symptoms" and "unless symptoms are severe" |
| **STOPPD10** | Neuroleptics as hypnotics, unless sleep disorder is due to psychosis or dementia | Y | Coded "sleep disorder due to psychosis" as the co-occurrence of sleep disorder and psychosis |
| **STOPPD11** | Acetylcholinesterase inhibitors with a known history of persistent bradycardia (< 60 beats/min.), heart block or recurrent unexplained syncope or concurrent treatment with drugs that reduce heart rate such as beta-blockers, digoxin, diltiazem, verapamil | Y | Did not code for "recurrent unexplained syncope" |
| **STOPPD12** | Phenothiazines as first-line treatment, since safer and more efficacious alternatives exist | N | Cannot code for "first-line" treatment so we don't know if other options were looked for. Also no other options are given as suggestions so we cannot check if they are used |
| **STOPPD13** | Levodopa or dopamine agonists for benign essential tremor | Y |  |
| **STOPPD14** | First-generation antihistamines | Y |  |
| **Renal system** |  | | |
| **STOPPE1** | Digoxin at a long-term dose greater than 125μg/day if eGFR < 30 ml/min/1.73m2 | N | Drug doses were not accessible |
| **STOPPE2** | Direct thrombin inhibitors (e.g. dabigatran) if eGFR < 30 ml/min/1.73m2 | Y | Use the clinical diagnosis instead of the eGFR values |
| **STOPPE3** | Factor Xa inhibitors (e.g. rivaroxaban, apixaban) if eGFR < 15 ml/min/1.73m2 | Y | Use the clinical diagnosis instead of the eGFR values |
| **STOPPE4** | NSAID’s if eGFR < 50 ml/min/1.73m2 | N | No clinical diagnosis uses threshold < 50 |
| **STOPPE5** | Colchicine if eGFR < 10 ml/min/1.73m2 | N | Clinical diagnosis threshold is < 15 |
| **STOPPE6** | Metformin if eGFR < 30 ml/min/1.73m2 | Y | Use the clinical diagnosis instead of the eGFR values |
| **Gastrointestinal system** |  | | |
| **STOPPF1** | Prochlorperazine or metoclopramide with Parkinsonism | Y |  |
| **STOPPF2** | PPI for uncomplicated peptic ulcer disease or erosive peptic oesophagitis at full therapeutic dosage for > 8 weeks | N | Drug doses were not accessible |
| **STOPPF3** | Drugs likely to cause constipation (e.g. antimuscarinic/anticholinergic drugs, oral iron, opioids, verapamil, aluminium antacids) in patients with chronic constipation where non-constipating alternatives are available | N | Cannot code or know whether non-constipating alternatives are available |
| **STOPPF4** | Oral elemental iron doses greater than 200 mg daily (e.g. ferrous fumarate> 600 mg/day, ferrous sulphate > 600 mg/day, ferrous gluconate> 1800 mg/day; no evidence of enhanced iron absorption above these doses). | N | Drug doses were not accessible |
| **Respiratory system** |  | | |
| **STOPPG1** | Theophylline as monotherapy for COPD | Y |  |
| **STOPPG2** | Systemic corticosteroids instead of inhaled corticosteroids for maintenance therapy in moderate-severe COPD | N | No ICD10 code to determine severity of COPD |
| **STOPPG3** | Anti-muscarinic bronchodilators (e.g. ipratropium, tiotropium) with a history of narrow angle glaucoma (may exacerbate glaucoma) or bladder outflow obstruction | Y | "Bladder outflow obstruction" we used prostatism and urinary retention |
| **STOPPG4** | Benzodiazepines with acute or chronic respiratory failure i.e. pO2 < 8.0 kPa ± pCO2 > 6.5 kPa | Y | Used the ICD10 code |
| **Musculoskeletal system** |  | | |
| **STOPPH1** | Non-steroidal anti-inflammatory drug (NSAID) other than COX-2 selective agents with history of peptic ulcer disease or gastrointestinal bleeding, unless with concurrent PPI or H2 antagonist | Y |  |
| **STOPPH2** | NSAID with severe hypertension (risk of exacerbation of hypertension) or severe heart failure | N | "severe" cannot be coded |
| **STOPPH3** | Long-term use of NSAID (>3 months) for symptom relief of osteoarthritis pain where paracetamol has not been tried | N | Cannot know whether paracetamol has been tried |
| **STOPPH4** | Long-term corticosteroids (>3 months) as monotherapy for rheumatoid arthrtitis | Y |  |
| **STOPPH5** | Corticosteroids (other than periodic intra-articular injections for mono-articular pain) for osteoarthritis | Y |  |
| **STOPPH6** | Long-term NSAID or colchicine (>3 months) for chronic treatment of gout where there is no contraindication to a xanthine-oxidase inhibitor (e.g. allopurinol, febuxostat) | N | Cannot code for contraindications |
| **STOPPH7** | COX-2 selective NSAIDs with concurrent cardiovascular disease | Y |  |
| **STOPPH8** | NSAID with concurrent corticosteroids without PPI prophylaxis | Y |  |
| **STOPPH9** | Oral bisphosphonates in patients with a current or recent history of upper gastrointestinal disease i.e. dysphagia, oesophagitis, gastritis, duodenitis, or peptic ulcer disease, or upper gastrointestinal bleeding | Y |  |
| **Urogenital system** |  | | |
| **STOPPI1** | Antimuscarinic drugs with dementia, or chronic cognitive impairment (risk of increased confusion, agitation) or narrow-angle glaucoma (risk of acute exacerbation of glaucoma), or chronic prostatism (risk of urinary retention). | Y |  |
| **STOPPI2** | Selective alpha-1 selective alpha blockers in those with symptomatic orthostatic hypotension or micturition syncope. | Y | Could only code part. Could not code for "symptomatic" orthostatic hypotension |
| **Endocrine system** |  | | |
| **STOPPJ1** | Sulphonylureas with a long duration of action (e.g. glibenclamide, chlorpropamide, glimepiride) with type 2 diabetes mellitus | Y |  |
| **STOPPJ2** | Thiazolidenediones (e.g. rosiglitazone, pioglitazone) in patients with heart failure | Y |  |
| **STOPPJ3** | Beta-blockers in diabetes mellitus with frequent hypoglycaemic episodes | N | Cannot code for hypoglycaemic episodes but could use hypoglycaemia ICD10 codes |
| **STOPPJ4** | Oestrogens with a history of breast cancer or venous thromboembolism | Y |  |
| **STOPPJ5** | Oral oestrogens without progestogen in patients with intact uterus | Y |  |
| **STOPPJ6** | Androgens (male sex hormones) in the absence of primary or secondary hypogonadism | Y |  |
| **Drugs that predictably increase the risk of falls in older people (FRIDs)** |  | | |
| **STOPPK1** | Benzodiazepines | Y |  |
| **STOPPK2** | Neuroleptic drugs | Y |  |
| **STOPPK3** | Vasodilator drugs (e.g. alpha-1 receptor blockers, calcium channel blockers, long-acting nitrates, ACE inhibitors, angiotensin I receptor blockers, ) with persistent postural hypotension i.e. recurrent drop in systolic blood pressure ≥ 20mmHg | Y |  |
| **STOPPK4** | Hypnotic Z-drugs e.g. zopiclone, zolpidem, zaleplon | Y |  |
| **Analgesic drugs** |  | | |
| **STOPPL1** | Use of oral or transdermal strong opioids (morphine, oxycodone, fentanyl, buprenorphine, diamorphine, methadone, tramadol, pethidine, pentazocine) as first line therapy for mild pain | N | Cannot code for first line therapy |
| **STOPPL2** | Use of regular (as distinct from PRN) opioids without concomitant laxative | Y |  |
| **STOPPL3** | Long-acting opioids without short-acting opioids for break-through pain | N | Cannot code for "break-through" pain |
| **Antimuscarinic / anticholinergic drug burden** |  | | |
| **STOPPM1** | Concomitant use of two or more drugs with antimuscarinic/anticholinergic properties (e.g. bladder antispasmodics, intestinal antispasmodics, tricyclic antidepressants, first generation antihistamines) | Y |  |
| **START Criteria** | | | |
| **Cardiovascular system** |  | | |
| **STARTA1** | Vitamin K antagonists or direct thrombin inhibitors or factor Xa inhibitors in the presence of chronic atrial fibrillation. | Y |  |
| **STARTA2** | Aspirin (75 mg – 160 mg once daily) in the presence of chronic atrial fibrillation, where Vitamin K antagonists or direct thrombin inhibitors or factor Xa inhibitors are contraindicated. | Y | The drug dose was disregarded and the prescribing of aspirin was assessed to identify the proportion of AF patients without antiplatelet and anticoagulant prescriptions, regardless of contraindications |
| **STARTA3** | Antiplatelet therapy (aspirin or clopidogrel or prasugrel or ticagrelor) with a documented history of coronary, cerebral or peripheral vascular disease | Y |  |
| **STARTA4** | Antihypertensive therapy where systolic blood pressure consistently > 160 mmHg and/or diastolic blood pressure consistently >90 mmHg; if systolic blood pressure > 140 mmHg and /or diastolic blood pressure > 90 mmHg, if diabetic. | N | No variable for measurement blood pressure |
| **STARTA5** | Statin therapy with a documented history of coronary, cerebral or peripheral vascular disease, unless the patient’s status is end-of-life or age is > 85 years. | Y | Can use ICD10 code for "palliative care" for end of stage |
| **STARTA6** | Angiotensin Converting Enzyme (ACE) inhibitor with systolic heart failure and/or documented coronary artery disease | Y |  |
| **STARTA7** | Beta-blocker with ischaemic heart disease. | Y |  |
| **STARTA8** | Appropriate beta-blocker (bisoprolol, nebivolol, metoprolol or carvedilol) with stable systolic heart failure | N | Cannot code for "stable" heart failure |
| **Respiratory system** |  | | |
| **STARTB1** | Regular inhaled B2 agonist or antimuscarinic bronchodilator (e.g. ipratropium, tiotropium) for mild to moderate asthma or COPD | Y |  |
| **STARTB2** | Regular inhaled corticosteroid for moderate-severe asthma or COPD, where FEV1 <50% of predicted value and repeated exacerbations requiring treatment with oral corticosteroids | N | There is no FEV value available so cannot code it |
| **STARTB3** | Home continuous oxygen with documented chronic hypoxaemia (i.e. pO2 < 8.0 kPa or 60 mmHg or SaO2 < 89%) | Y |  |
| **Central nervous system & eyes** |  | | |
| **STARTC1** | L-DOPA or a dopamine agonist in idiopathic Parkinson’s disease with functional impairment and resultant disability. | N | ICD10 code does not distinguish between idiopathic and primary parkinson's disease |
| **STARTC2** | Non-TCA antidepressant drug in the presence of persistent major depressive symptoms. | Y | "Persistent major depressive symptoms" could use depression or "major depressive disorder" |
| **STARTC3** | Acetylcholinesterase inhibitor (e.g. donepezil, rivastigmine, galantamine) for mild-moderate Alzheimer’s dementia or Lewy Body dementia (rivastigmine). | Y | Could only assess in the case of Lewy Body dementia. ICD10 code does not cover severity of Alzheimer |
| **STARTC4** | Topical prostaglandin, prostamide or beta-blocker for primary open-angle glaucoma. | Y |  |
| **STARTC5** | Selective serotonin reuptake inhibitor (or SNRI or pregabalin if SSRI contraindicated) for persistent severe anxiety that interferes with independent functioning. | Y |  |
| **STARTC6** | Dopamine agonist (ropinirole or pramipexole or rotigotine) for Restless Legs Syndrome, once iron deficiency and severe renal failure have been excluded. | Y | Can use ICD10 code and severe renal failure defined as eGFR > 30 ml/min/m2 |
| **Gastrointestinal system** |  | | |
| **STARTD1** | Proton Pump Inhibitor with severe gastro-oesophageal reflux disease or peptic stricture requiring dilatation. | N | Cannot code for "severe" reflux disease or peptic stricture requiring dilation |
| **STARTD2** | Fibre supplements (e.g. bran, ispaghula, methylcellulose, sterculia) for diverticulosis with a history of constipation. | Y |  |
| **Musculoskeletal system** | 1 | | |
| **STARTE1** | Disease-modifying anti-rheumatic drug (DMARD) with active, disabling rheumatoid disease. | N | Cannot code for "active and disabling" |
| **STARTE2** | Bisphosphonates and vitamin D and calcium in patients taking long-term systemic corticosteroid therapy. | Y |  |
| **STARTE3** | Vitamin D and calcium supplement in patients with known osteoporosis and/or previous fragility fracture(s) and/or (Bone Mineral Density T-scores more than -2.5 in multiple sites). | Y | Can code for osteoporosis and fractures but not Bone Mineral Density T score |
| **STARTE4** | Bone anti-resorptive or anabolic therapy (e.g. bisphosphonate, strontium ranelate, teriparatide, denosumab) in patients with documented osteoporosis, where no pharmacological or clinical status contraindication exists (Bone Mineral Density T-scores -> 2.5 in multiple sites) and/or previous history of fragility fracture(s). | Y | Could code part of the criterion. It was not possible to code for "where no pharmacological or clinical status contraindication exists" |
| **STARTE5** | Vitamin D supplement in older people who are housebound or experiencing falls or with osteopenia (Bone Mineral Density T-score is > -1.0 but < -2.5 in multiple sites). | Y | The criterion was partially codable. Housebound could not be coded. |
| **STARTE6** | Xanthine-oxidase inhibitors (e.g. allopurinol, febuxostat) with a history of recurrent episodes of gout. | Y | Define recurrent episodes of gout as chronic gout |
| **STARTE7** | Folic acid supplement in patients taking methotrexate. | Y |  |
| **Endocrine system** |  | | |
| **STARTF1** | ACE inhibitor or Angiotensin Receptor Blocker (if intolerant of ACE inhibitor) in diabetes with evidence of renal disease i.e. dipstick proteinuria or microalbuminuria (>30mg/24 hours) with or without serum biochemical renal impairment. | Y | Don't have dipstick proteinuria or microalbuminuria but can use ICD10 codes |
| **Urogenital system** | 1 | | |
| **STARTG1** | Alpha-1 receptor blocker with symptomatic prostatism, where prostatectomy is not considered necessary. | N | No ICD10 code for "where prostatectomy is not considered necessary" |
| **STARTG2** | 5-alpha reductase inhibitor with symptomatic prostatism, where prostatectomy is not considered necessary. | N | No ICD10 code for "where prostatectomy is not considered necessary" |
| **STARTG3** | Topical vaginal oestrogen or vaginal oestrogen pessary for symptomatic atrophic vaginitis. | N | Cannot code for symptomatic |
| **Analgesics** |  | | |
| **STARTH1** | High-potency opioids in moderate-severe pain, where paracetamol, NSAIDs or low-potency opioids are not appropriate to the pain severity or have been ineffective. | N | Cannot code for pain severity and appropriateness of drug to pain severity |
| **STARTH2** | Laxatives in patients receiving opioids regularly. | Y |  |
| **Vaccines** |  | | |
| **STARTI1** | Seasonal trivalent influenza vaccine annually | N | Vaccine information was not accessible |
| **STARTI2** | Pneumococcal vaccine at least once after age 65 according to national guidelines | N | Vaccine information was not accessible |
