# Supplementary material for: Potentially inappropriate prescribing in polymedicated older adults with atrial fibrillation and multimorbidity: a Swedish national register-based cohort study
Source: Front Pharmacol. 2024 Sep 10;15:1476464. doi: 10.3389/fphar.2024.1476464 (PMC11420530; doi:10.3389/fphar.2024.1476464)
Supplement: Supplementary file 4 [file DataSheet5.docx]

**Potentially inappropriate prescribing in polymedicated older adults with atrial fibrillation and multimorbidity: A Swedish national register-based cohort study**

Cheima Amrouch^1,2^, Davide Liborio Vetrano^3,4^, Cecilia Damiano^5^, Lu Dai^3^, Amaia Calderón-Larrañaga^3,4^, Maxim Grymonprez^2,6^, Marco Proietti^7,8^, Gregory Y.H. Lip^9,10^, Søren P. Johnsen^10^, Jonas W. Wastesson^3,11^, Kristina Johnell^11^, Delphine De Smedt^1^*, Mirko Petrovic^2^*, *on behalf of the AFFIRMO project
** Shared last-author

*1* Department of Public Health and Primary Care, Ghent University, Ghent, Belgium
*2* Department of Internal Medicine and Paediatrics, Ghent University, Ghent, Belgium
*3* Aging Research Center, Department of Neurobiology, Care Sciences and Society, Karolinska Institutet and Stockholm University, Stockholm, Sweden
*4* Stockholm Gerontology Research Center, Stockholm, Sweden
*5* Department of Cardiovascular, Endocrine-Metabolic Diseases and Aging, Istituto Superiore di Sanità, Rome, Italy
*6* Department of Bioanalysis, Pharmaceutical Care Unit, Ghent University, Ghent, Belgium
*7* Department of Clinical Sciences and Community Health, University of Milan, Milan, Italy
*8* Division of Subacute Care, IRCCS Istituti Clinici Scientifici Maugeri, Milan, Italy
*9* Liverpool Centre for Cardiovascular Science at University of Liverpool, Liverpool John Moores University and Liverpool Heart & Chest Hospital, Liverpool, UK
*10* Danish Center for Health Services Research, Department of Clinical Medicine, Aalborg University, Aalborg, Denmark
*11* Department of Medical Epidemiology and Biostatistics, Karolinska Institutet, Stockholm, Sweden

**AFFIRMO consortium members:**

Søren Påske Johnsen, Pia Cordsen (Aalborg Universitet), Gregory Lip, Riccardo Proietti, Deirdre Lane, Martin O’Flaherty, Carrol Gamble, Iain Buchan, Christodoulos Kypridemos, Brendan Collins, Donato Leo (The University of Liverpool), Mirko Petrovic, Delphine De Smedt, Cheima Amrouch (Universiteit Gent), Davide Liborio Vetrano, Amaia Calderón-Larrañaga, Lu Dai (Karolinska Institutet), Stefania Maggi, Marianna Noale (Consiglio Nazionale delle Ricerche), Gheorghe-Andrei DAN, Anca Rodica Dan, Elisabeta Badila, Adrian Catalin Buzea, Raluca Popescu Universitatea de Medicina si Farmacie “Carol Davila” din Bucuresti), Nicola Ferri, Alessandra Buja, Giuseppe Sergi, Vincenzo Stefano Rebba, Caterina Trevisan (Università degli Studi di Padova), Tatjana Potpara (Faculty of Medicine, University of Belgrade), Laura Vivani, Silvia Ananstasia (Moverim Consulting sprl), Alessandro Ferri, Gehad Shehata, Nadia Rosso, Marco Cicerone (Advice Pharma Group srl), Jacek Marczyk (Ontonix), Trudie Lobban (Arrhythmia Alliance), Georg Ruppe (European Union Geriatric Medicine Society aisbl), Benedetta Marcozzi, Federica Censi, Robero Da Cas, Cecilia Damiano (Istituto Superiore di Sanità), Guendalina Graffigna, Caterina Bosio, Lorenzo Palamenghi, Serena Barello (Università Cattolica del Sacro Cuore), Marco Proietti (University of Milan), Aldo Pietro Maggioni, Andrea Lorimer, Donata Lucci (Heart Care Foundation Onlus), Dipak Kalra, Nathan Lea (The European Institute for Innovation through Health Data), John Ainsworth, Charlotte Stockton-Powdrell, Alam Sanaullah (The University of Manchester), Francisco Marín Ortuño, José Miguel Rivera-Caravaca University of Manchester), Francisco Marín Ortuño, José Miguel Rivera-Caravaca, Vanessa Roldán, María Asunción Esteve-Pastor, Cecilia López-García, Pablo Gil-Pérez (Universidad De Murcia), Mariya Tokmakova (Medical University Plovdiv).
