# Supplementary material for: Potentially inappropriate prescribing in polymedicated older adults with atrial fibrillation and multimorbidity: a Swedish national register-based cohort study
Source: Front Pharmacol. 2024 Sep 10;15:1476464. doi: 10.3389/fphar.2024.1476464 (PMC11420530; doi:10.3389/fphar.2024.1476464)
Supplement: Supplementary file 5 [file DataSheet1.docx]

**Potentially inappropriate prescribing in polymedicated older adults with atrial fibrillation and multimorbidity:**

**A Swedish national register-based cohort study**

Cheima Amrouch^1,2^, Davide Liborio Vetrano^3,4^, Cecilia Damiano^5^, Lu Dai^3^, Amaia Calderón-Larrañaga^3,4^, Maxim Grymonprez^2,6^, Marco Proietti^7,8^, Gregory Y.H. Lip^9,10^, Søren P. Johnsen^10^, Jonas W. Wastesson^3,11^, Kristina Johnell^11^, Delphine De Smedt^1^*, Mirko Petrovic^2^*, *on behalf of the AFFIRMO project
** Shared last-author

*1* Department of Public Health and Primary Care, Ghent University, Ghent, Belgium
*2* Department of Internal Medicine and Paediatrics, Ghent University, Ghent, Belgium
*3* Aging Research Center, Department of Neurobiology, Care Sciences and Society, Karolinska Institutet and Stockholm University, Stockholm, Sweden
*4* Stockholm Gerontology Research Center, Stockholm, Sweden
*5* Department of Cardiovascular, Endocrine-Metabolic Diseases and Aging, Istituto Superiore di Sanità, Rome, Italy
*6* Department of Bioanalysis, Pharmaceutical Care Unit, Ghent University, Ghent, Belgium
*7* Department of Clinical Sciences and Community Health, University of Milan, Milan, Italy
*8* Division of Subacute Care, IRCCS Istituti Clinici Scientifici Maugeri, Milan, Italy
*9* Liverpool Centre for Cardiovascular Science at University of Liverpool, Liverpool John Moores University and Liverpool Heart & Chest Hospital, Liverpool, UK
*10* Danish Center for Health Services Research, Department of Clinical Medicine, Aalborg University, Aalborg, Denmark
*11* Department of Medical Epidemiology and Biostatistics, Karolinska Institutet, Stockholm, Sweden

**Operationalisation of cardiovascular mortality ICD-10 codes:**

*I0, I10, I11, I12, I13, I15, I2, I3, I4, I50, I51, I6, I7*

**Operationalisation of cardiovascular hospitalisation ICD-10 codes:**

*I10, I20, I21***,**  *I22, I23, I24, I25, I26, I312, I34, I35, I50, I60, I61, I62, I63, I64, I73, I74, I864A , G45, D62, R31, R04, D500, H313, H356, H431, H450, I850, K250, K252, K254, K256, K260, K262, K264, K266, K270, K272, K274, K276, K280, K282, K284, K286, K625, K661, K920, K921, K922, S064, S065, S066, J942, K228F, K298A, K638B, K638C, K838F, K868G, H052A, S368D, G951A, Z941, Z95, I48*

**Operationalisation of stroke ICD-10 codes:**

*I63, I64*

**Operationalisation of bleeding ICD-10 codes:**

*D62, I60, I61, I62, R31, R04, D500, H313, H356, H431, H450, I312, I850, K250, K252, K254, K256, K260, K262, K264, K266, K270, K272, K274, K276, K280, K282, K284, K286, K625, K661, K920, K921, K922, S064, S065, S066, J942, K228F, K298A, K638B, K638C, K838F, K868G, I864A, H052A, S368D, G951A*

**Operationalisation of injurious falls ICD-10 codes:**

*W00, W01, W05, W06, W07, W08, W09, W10, W17, W18, W19*
